# Novel alleles of rice *eIF4G* generated by CRISPR/Cas9‐targeted mutagenesis confer resistance to *Rice tungro spherical virus*

**DOI:** 10.1111/pbi.12927

**Published:** 2018-04-30

**Authors:** Anca Macovei, Neah R. Sevilla, Christian Cantos, Gilda B. Jonson, Inez Slamet‐Loedin, Tomáš Čermák, Daniel F. Voytas, Il‐Ryong Choi, Prabhjit Chadha‐Mohanty

**Affiliations:** ^1^ Genetics and Biotechnology Division International Rice Research Institute (IRRI) Metro Manila Philippines; ^2^ Department of Genetics Cell Biology & Development and Center for Genome Engineering University of Minnesota Minneapolis MN USA; ^3^ Present address: Department of Biology and Biotechnology ‘L. Spallanzani’ University of Pavia Pavia Italy; ^4^ Present address: Huck Institute of the Life Sciences Pennsylvania State University University Park PA USA

**Keywords:** rice, eIF4G, CRISPR/Cas9, rice tungro spherical virus, mutagenesis

## Abstract

Rice tungro disease (RTD) is a serious constraint in rice production across tropical Asia. RTD is caused by the interaction between *Rice tungro spherical virus* (RTSV) and *Rice tungro bacilliform virus*. RTSV resistance found in traditional cultivars has contributed to a reduction in the incidence of RTD in the field. Natural RTSV resistance is a recessive trait controlled by the translation initiation factor 4 gamma gene (eIF4G). The Y^1059^V^1060^V^1061^ residues of eIF4G are known to be associated with the reactions to RTSV. To develop new sources of resistance to RTD, mutations in *eIF4G* were generated using the CRISPR/Cas9 system in the RTSV‐susceptible variety IR64, widely grown across tropical Asia. The mutation rates ranged from 36.0% to 86.6%, depending on the target site, and the mutations were successfully transmitted to the next generations. Among various mutated *eIF4G* alleles examined, only those resulting in in‐frame mutations in SVLFPNLAGKS residues (mainly NL), adjacent to the YVV residues, conferred resistance. Furthermore, our data suggest that *eIF4G* is essential for normal development, as alleles resulting in truncated eIF4G could not be maintained in homozygous state. The final products with RTSV resistance and enhanced yield under glasshouse conditions were found to no longer contain the Cas9 sequence. Hence, the RTSV‐resistant plants with the novel *eIF4G* alleles represent a valuable material to develop more diverse RTSV‐resistant varieties.

## Introduction

Achieving high crop yields with efficient and sustainable agricultural practices is essential to attain global food security. Within the current context of climate change affecting the arable farm land and natural resources, the demand to produce sufficient agricultural products for the increasing human population is constantly expanding (Ronald, 2014). The escalation of biotic and abiotic stresses adds even more pressure to agricultural crop production. This especially applies in the case of rice as a species responsible for feeding billions of people, many of whom represent the low‐income population from developing and underdeveloped countries in Asia and Africa (Seck *et al*., 2012).

The effort to increase rice production is often constrained by outbreaks of viral diseases. Among these, rice tungro disease (RTD) causes severe disruption of rice production, affecting more than 350 000 ha throughout the main rice‐producing Asian countries (Azzam and Chancellor, 2002; Chancellor *et al*., 2006; Muralidharan *et al*., 2003). Rice plants affected by RTD show symptoms such as stunting or yellow discoloration of the leaves at the early stages, and reduced tillering and sterile panicles at later stages (Hull, 1996). RTD is caused by the interaction between *Rice tungro spherical virus* (RTSV), having a single‐stranded RNA genome, and *Rice tungro bacilliform virus* (RTBV), having a double‐stranded DNA genome (Hull, 1996). RTSV and RTBV are transmitted predominantly by green leafhopper (GLH) species such as *Nephotettix virescens* and *N. nigropictus* (Hibino and Cabauatan, 1987). RTBV is responsible for the development of the disease symptoms, while RTSV acts as a helper virus assisting transmission of RTBV by GLH and enhancing the symptoms (Hull, 1996). Cultivars with resistance to RTSV can contribute to a reduction in RTD in large fields, as plants infected with RTBV alone do not serve as sources for secondary spread (Anjaneyulu *et al*., 1994).

Extensive breeding programmes, conducted at International Rice Research Institute (IRRI), based on the screening of rice germplasm collections led to the identification of a number of rice cultivars resistant to RTD (Hibino *et al*., 1990; Khush *et al*., 2004; Sebastian *et al*., 1996). Several studies then focused on the Indica rice cultivar Utri Merah, which is resistant to both RTSV and RTBV (Azzam *et al*., 2001; Cabunagan *et al*., 1999; Ebron *et al*., 1994). Using near‐isogenic lines (NILs) derived from Utri Merah, Encabo *et al*. (2009) demonstrated that the resistance to RTSV and RTBV in Utri Merah is an independent trait. RTSV resistance was found to be a recessive trait controlled by the translation initiation factor 4 gamma (*eIF4G*) gene (Lee *et al*., 2010). By comparing the gene sequences in RTSV‐resistant and RTSV‐susceptible cultivars, the authors pinpointed that the single‐nucleotide polymorphisms (SNPs) or deletion affecting the Y^1059^V^1060^V^1061^ amino acid residues were responsible for the resistant phenotype (Lee *et al*., 2010). Another report showed the involvement of *eIF(iso)4G* in recessive resistance to *Rice yellow mottle virus* (RYMV) (Albar *et al*., 2006). The association of *eIF4G* and *eIF(iso)4G* with virus resistance is due to their essential roles in the assembly of basal translational initiation factors at the 5′‐end of mRNA as well as in the cap‐independent translation of viral RNA genomes (Kneller *et al*., 2006; Sonenberg and Hinnebusch, 2009). To undergo translation for synthesis of viral proteins, RNA viruses exploit the host cellular translational machinery to reprogramme translation to favour their own protein synthesis (Dreher and Miller, 2006).

In the past, traditional breeding techniques had been used to introduce various disease resistance genes from natural variants into valuable varieties. Nowadays, genome editing tools based on the activity of site‐specific nucleases greatly facilitate the introduction of targeted mutations at designated genomic sites that are associated with disease resistance traits (Lee *et al*., 2016; Zhu *et al*., 2017). Among such genome editing tools, the CRISPR/Cas9 (clustered regularly interspaced short palindromic repeat‐associated protein 9) system is gaining the highest interest, as it is the most user‐friendly and highly efficient (Barakate and Stephens, 2016; Belhaj *et al*., 2013). With CRISPR/Cas9 targeted cleavage, small deletions or insertions are introduced at specific sites by harnessing the ability of nonhomologous end‐joining (NHEJ) to repair the induced double‐strand breaks (Belhaj *et al*., 2013). The CRISPR/Cas9 system has been successfully used to engineer virus resistance in plants by targeting either the viral genome or the host genome (Zaidi *et al*., 2016). When considering the plant host genome, genes for translation initiation factors *eIF4E* and *eIF(iso)4E* were mutated using the CRISPR/Cas9 system, resulting in the development of *Arabidopsis* lines resistant to *Turnip mosaic virus* (Pyott *et al*., 2016), and cucumber plants with broad‐spectrum resistance to *Cucumber vein yellowing virus*,* Zucchini yellow mosaic virus* and *Papaya ringspot mosaic virus* (Chandrasekaran *et al*., 2016).

In this study, the CRISPR/Cas9 system was used to mutate the *eIF4G* gene in *Oryza sativa* var. *indica* cv. IR64, a widely grown cultivar across tropical Asia. IR64 is susceptible to RTSV (Hibino *et al*., 1988), possessing a susceptible (S)‐type allele of *eIF4G*, whereas resistant cultivars able to suppress RTSV infection have the non‐S‐type allele of *eIF4G* with the nonsynonymous SNPs or deletion causing mutations at the Y^1059^V^1060^V^1061^ residues (Lee *et al*., 2010). Hence, we designed CRISPR/Cas9 reagents to target the sequences flanking this region of *eIF4G* with the aim to develop RTSV‐resistant varieties that could potentially be released as nongenetically modified plants to the market whether/when proper regulatory policies are set in place. Our results showed that targeted mutagenesis mediated by CRISPR/Cas9 was highly efficient and dependent on the gRNA sequence, and the targeted mutations were inherited in subsequent generations. Importantly, some of the edited plants were resistant to RTSV, and the analysis of novel alleles of *eIF4G* (from the sequence SVLFPNLAGKS adjacent to the YVV residues) in the RTSV‐resistant plants suggested that amino acid residues NL are also associated with RTSV resistance. The selected T_2_ plants were found to no longer contain the Cas9, and no off‐target effects were evidenced. When the T_2_ plants were inoculated with RTSV, their yield was significantly higher than that of wild‐type IR64 under glasshouse conditions.

## Results

### CRISPR/Cas9 efficiently generates variation in the rice *eIF4G* in T_0_ generation

**Figure 1:**
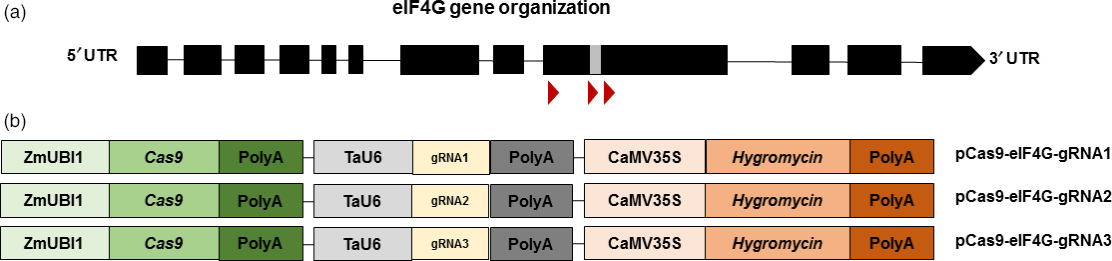
Gene editing of rice *eIF4G*. (a) Schematic illustration of the *eIF4G* locus (LOC_Os07g36940) composed of 12 exons (black boxes) and 11 introns (black lines); the YVV residues known to be associated with reactions to RTSV are indicated with a grey box and the gRNA target sequences are represented as red triangles. (b) Schematic diagrams of CRISPR/Cas9 vectors. TaU6, *Triticum aestivum* U6 promoter; ZmUBI1, *Zea mays* ubiquitin promoter; Cas9, CRISPR‐associated protein‐9 nuclease gene; PolyA, termination signal; CaMV35S, *Cauliflower mosaic virus* 35S promoter; Hygromycin, hygromycin resistance gene.

To construct the CRISPR/Cas9 vectors, three gRNAs’ targeting sequences in the region between nucleotide positions 4140 and 4414 of *eIF4G* (Figure 1a, Table S1) were designed. The target locations were chosen based on the previous study showing that SNPs at positions 4387 and 4390 were associated with the reaction to RTSV (Lee *et al*., 2010). The gRNA1 was designed to target around 230 bp upstream of the indicated SNPs, gRNA2 was designed to target 10 bp upstream of this region, and gRNA3 was designed to target 5 bp downstream of the SNP^4390^.

An *in silico* analysis was performed on the DNA sequences of the three gRNAs to evidence the GC% and types of DNA bases in important positions (N‐20 and N‐3) (Liu *et al*., 2016) (Table S1). The GC contents (45%–55%) were in the accepted range (30%–80%) for plant‐derived gRNAs (Liang *et al*., 2016). T at position N‐3 and C at position N‐20 were associated with increased cutting efficiency in animal cells (Liu *et al*., 2016), but not in plants (Liang *et al*., 2016). In our gRNAs, all three sequences possess T at position N‐3, while C at position N‐20 is present only in gRNA2, as the gRNA1 and gRNA3 have G at this position (Table S1).

**Figure 2:**
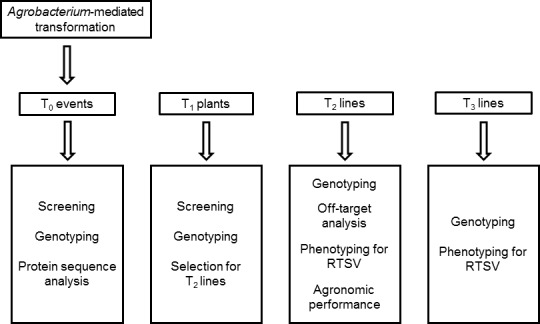
Flow chart of the experiments conducted. CRISPR/Cas9 constructs were introduced into rice immature embryos through *Agrobacterium*‐mediated transformation. Following *in vitro* tissue culture, the regenerated T_0_ plants were grown in the IRRI glasshouse facility. Mutation screening, genotyping and protein sequence analyses were performed in the T_0_ population. Selected events were propagated through seeds to generate the T_1_ population which was subsequently screened and genotyped. Then, the T_2_ and T_3_ populations were generated and analysed for their genotypes for *eIF4G* and phenotypes for RTSV infection.

The T‐DNA in the final vectors pCas9‐eIF4G‐gRNA1, pCas9‐eIF4G‐gRNA2 and pCas9‐eIF4G‐gRNA3 contained three cassettes: (i) the Cas9 cassette to express a plant codon‐optimized Cas9 from *Streptococcus pyogenes* driven by the *Zea mays* ubiquitin (ZmUBI1) promoter, (ii) the gRNA cassette to express one of the three gRNAs, each driven by the *Triticum aestivum* U6 promoter, and (iii) the plant selection cassette containing *hyg* (conferring resistance to hygromycin) driven by the CaMV35S promoter (Figure 1b). The entire experimental scheme pursued after the vector construction is represented in Figure 2.

**Figure 3:**
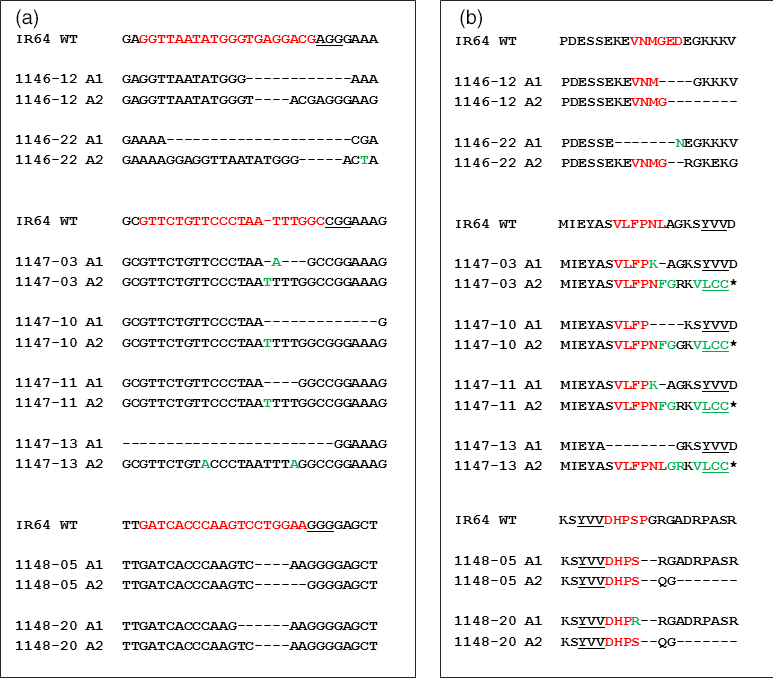
Selective T_0_ events with detected mutations and corresponding changes at the protein level. (a) Mutations detected in selected T_0_ events generated from constructs pCas9‐eIF4G‐gRNA1 (1146 events), pCas9‐eIF4G‐gRNA2 (1147 events) and pCas9‐eIF4G‐gRNA3 (1148 events). Sequences in red represent the gRNA targets, and the PAM sequences are underlined. Deletions are indicated by ‘‐’, and insertions are shown in green. (b) Protein sequence alignment between eIF4G of IR64, and mutated T_0_ events. The protein sequence alignment was performed using COBALT multiple alignment tool (https://www.ncbi.nlm.nih.gov/tools/cobalt/re_cobalt.cgi). The protein sequences corresponding to gRNA targets are indicated in red. Amino acid changes caused by mutations are shown in green. Missing amino acids are represented as ‘‐’. The YVV sequence is presented in bold underlined letters.

*Agrobacterium*‐mediated transformation was performed using rice immature embryos, and the generated events were designated as 1146 when generated with pCas9‐eIF4G‐gRNA1, 1147 when generated with pCas9‐eIF4G‐gRNA2 and 1148 when generated with pCas9‐eIF4G‐gRNA3. The T_0_ events selected on hygromycin‐containing media were first examined for the presence of T‐DNA by PCR, and the overall transformation efficiency was 48.0% (Table 1). Subsequently, the T7 endonuclease I (T7EI) assay was performed to screen for the presence of mutations (Figure S1). Of the 72 transgenic events, 43 (59.7%) were positive for mutations in the T7EI assay (Table 1). The mutation rate for the individual constructs was 36.0% (pCas9‐eIF4G‐gRNA1), 86.8% (pCas9‐eIF4G‐gRNA2) and 65.6% (pCas9‐eIF4G‐gRNA3) (Table 1). All 43 events with putative mutations underwent sequencing analysis, and the observed types of mutations are presented in Table S2. The majority of the mutations in the T_0_ events were biallelic (27 of 43, 62.8%) or chimeras (15 of 43, 34.8%). Our results are similar to those from previous studies in rice CRISPR/Cas9‐induced mutagenesis (Li *et al*., 2017; Miao *et al*., 2013; Xu *et al*., 2015; Zhang *et al*., 2014). The event with the homozygous mutation (1148‐16, with a five‐nucleotide deletion) detected via sequencing analysis proved to be sterile (data not shown). Different types of mutations are present in the individual events, with most of them possessing more than just one type of mutation (Table S2). The most frequent were deletions (83.3%–100%), but substitutions (25.0%–45.0%) and insertions (5.0%–58.3%) were also present (Table S3). When considering each type of mutation, 16 (37.2%) T_0_ events contained just deletions, whereas 15 (34.8%) events contained a combination of deletions and substitutions. Moreover, three (6.9%) events (1148‐06, 1147‐03 and 1147‐09) had a combination of deletions, substitutions and insertions, while no event with just insertions or just substitutions was obtained (Table S2). The number of bases affected ranged from 1 to 3 for insertions and substitutions, and from 1 to 63 for deletions, with more than 50% of the events showing deletions longer than 5 bp (Table S2). These observations are in line with the previous studies performed in rice (Li *et al*., 2017; Miao *et al*., 2013; Xu *et al*., 2015; Zhang *et al*., 2014), although we obtained higher rates of insertions and substitutions. Alignments of the targeted sequence in IR64 and mutated sequences in selected T_0_ events are presented in Figure 3a and selective chromatograms are provided in Figure S2.

**Table 1:** Transformation efficiency and mutation rate in T_0_ events generated by the *Agrobacterium*‐mediated transformation of immature embryos with CRISPR/Cas9 constructs

Vector ID	Vector description	No. of immature embryos	No. of regenerated plants	No. of transgenic events	Transformation efficiency (%)	No. of events with mutations	Mutation rate (%)
1146	pCas9‐eIF4G‐gRNA1	50	25	25	50.0	9	36.0
1147	pCas9‐eIF4G‐gRNA2	50	15	15	30.0	13	86.6
1148	pCas9‐eIF4G‐gRNA3	50	32	32	64.0	21	65.6
Total		150	72	72	48.0	43	59.7

**Figure 4:**
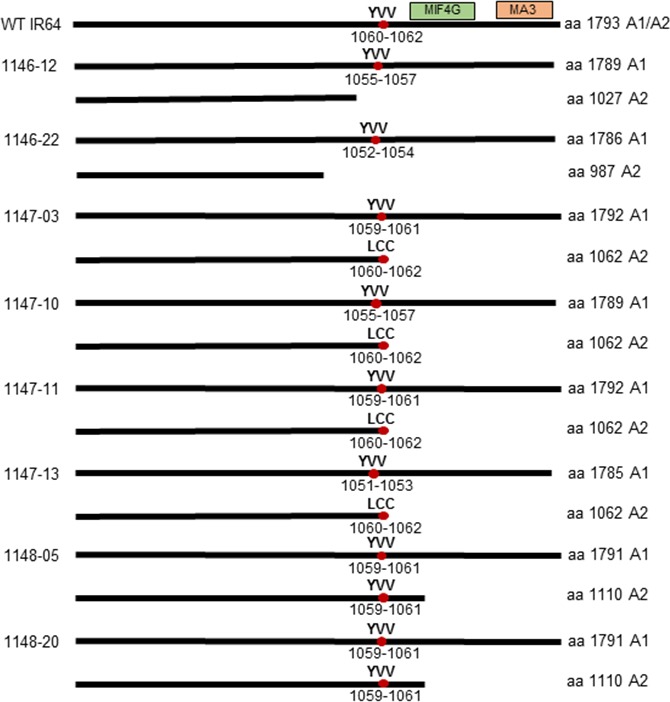
Schematic representation of selective mutated eIF4G proteins in T_0_ events. The eIF4G of IR64 is composed of 1793 amino acids. The highly conserved MIF4G and MA3 domains are indicated by the green and orange boxes, respectively. The YVV residues indicated are those known to be associated with reactions to RTSV. The numbers of amino acids in the predicted proteins are indicated to the right.

The predicted protein sequences from the mutated *eIF4G* alleles were determined in selected T_0_ events to evaluate the effect of mutations at this level. All of the selected events have one allele (A1) predicted to encode eIF4G of nearly full length with one to seven amino acids deleted, while the second allele (A2) is predicted to encode a truncated eIF4G (Figures 3b, 4). The predicted truncated eIF4G variants can be divided into three categories: (i) truncated upstream of the YVV residues (events 1146‐12, 1146‐22), (ii) truncated with YVV residues mutated to LCC (events 1147‐03, 1147‐10, 1147‐11, 1147‐13) and (iii) truncated downstream of the YVV residues (events 1148‐05, 1148‐20) (Figure 4). Events 1146 were generated with pCas9‐eIF4G‐gRNA1, which targets the nucleotides translated to the VNMGED sequence (amino acids 1008‐1013) located 76 amino acids upstream of the YVV sequence (amino acids 1060‐1062). The predicted protein sequence in events 1146‐12 and 1146‐22 shows that the VNMGED sequence was mutated in both alleles (Figure 3b). The predicted products from one of the alleles in both events were truncated upstream the YVV sequence (Figure 4). In the events generated with pCas9‐eIF4G‐gRNA2 construct (1147), gRNA2 targets the nucleotides translated to the VLFPNL sequence (amino acids 1050‐1155) located five amino acids upstream of the YVV sequence. The predicted protein sequences showed mutated amino acid residues within or between the SVLFPNLAGKSYVV sequence (mainly the NL residues) in one allele, while the predicted proteins in the second allele were truncated and had the YVV sequence mutated to LCC (Figure 3b). Lastly, in the events generated with pCas9‐eIF4G‐gRNA3 construct (1148), gRNA3 targets nucleotides translated to the DHPSPG sequence (amino acids 1063‐1069) located immediately downstream of the YVV sequence. Disruption of the DHPSPG sequence was observed in the products predicted from both alleles in two T_0_ events, 1148‐5 and 1148‐20, but the YVV sequence in the predicted product remained intact (Figure 3b). The infertile T_0_ event 1148‐16 contains homozygous *eIF4G* alleles with five nucleotides deleted. This deletion was predicted to result in mutations of DHPSPG to DHPRKG without affecting the YVV sequence, but creating a premature stop codon immediately after DHPRKG (Figure S3). This suggests that eIF4G is essential for plant growth as plants in which both *eIF4G* alleles produced truncated proteins had sterile seeds, while the presence of an allele encoding a nearly full‐length eIF4G with localized mutations seemed to be sufficient to sustain normal plant growth and seed development.

### Novel *eIF4G* alleles are transmitted to T_1_ and T_2_ generations with no detectable mutations in the closest off‐target site

Several T_1_ progenies generated from selected T_0_ events underwent a T7EI assay and genotyping analyses to investigate the pattern of transmission of CRISPR/Cas9‐mediated targeted gene modifications. As expected for biallelic and chimeric T_0_ events containing T‐DNA, we observed that some mutations were lost in the T_1_ generation (e.g. 1146‐12 and 1147‐03), whereas some new mutations occurred as well (e.g. 1147‐13) (Table S4). Based on genotyping analyses, among the 24 T_1_ plants analysed, just one (1148‐05‐13) was homozygous, while the rest had biallelic mutations. The chimeric mutations found in T_0_ events were no longer present in T_1_ plants. The presence/absence of Cas9 was determined by PCR, and 17 of 24 events (70.8%) were negative, while 7 (29.2%) were positive for Cas9 (Table S4). The segregation ratios of mutations in T_1_ plants showed that generally these did not follow a Mendelian distribution (Table S5 **)**. Similar observations with regard to loss/acquisition of mutations and distorted inheritance pattern in the T_1_ population were also reported in previous studies (Pan *et al*., 2016; Xu *et al*., 2015).

Seeds from selected T_1_ plants with biallelic mutations and with or without the Cas9 transgene were sown to generate the T_2_ population. All T_2_ plants examined were negative for Cas9 (Table S4). The expected segregation pattern for biallelic mutations was 1 (homozygous for mutation 1) : 2 (biallelic) : 1 (homozygous for mutation 2) (Feng *et al*., 2014; Xu *et al*., 2015). However, one of the alleles was predominantly lost and most segregation ratios were 1:2:0 or 1:1:0 (Table S5). The distorted segregation of mutations in both T_1_ and T_2_ populations suggests that the two alleles are not inherited with equal frequencies (Pan *et al*., 2016; Zhang *et al*., 2014). One of the reasons for the distorted segregation could be that the alleles that were lost more often were those encoding a truncated protein, as the presence of functional eIF4G seems to be essential for plant development as addressed above.

As low‐frequency cases of off‐target cleavage by the CRISPR/Cas9 system have been reported in plants (Shan *et al*., 2013; Zhang *et al*., 2014), we examined the presence of potential off‐target mutations in selected T_2_ plants of the 1147 lines (pCas9‐eIF4G‐gRNA2 construct). We performed the analysis in the T_2_ plants as these no longer contain Cas9, and hence, no additional cuts would be generated. Potential off‐target loci were predicted based on the protospacer adjacent motif (PAM) sequence using the CRISPR‐P online tool (Lei *et al*., 2014). Three potential sites containing three to four mismatched bases were retrieved (Figure S4). We chose to further analyse the site with three mismatches (Chr11:17936212) representing the closest off‐target. DNA was extracted from 23 randomly selected T_2_ plants of the 1147 lines and subjected to PCR and sequencing analyses. No mutations were found in the putative off‐target site in any of the T_2_ plants examined (Figure S4), underlining the high specificity of CRISPR/Cas9‐induced mutagenesis.

### In‐frame mutations in *eIF4G* confer RTSV resistance in T_2_ and T_3_ population

T_2_ plants generated from selected T_1_ events were evaluated for RTSV resistance to assess the association between the *eIF4G* mutations and the reaction to RTSV. As plants at the early seedling stage are most susceptible to RTSV (Rao and Anjaneyulu, 1980), we inoculated 10‐day‐old T_2_ plants with RTSV via GLH for 24 h. The mutation type, zygosity, protein sequence alignment and reactions to RTSV of selected T_2_ plants are shown in Data S1. The results of enzyme‐linked immunosorbent assay (ELISA) using an RTSV‐specific antibody showed that all T_2_ plants generated from 1147 events obtained using the pCas9‐eIF4G‐gRNA2 construct were resistant to RTSV (Tables S4, S6). The T_2_ plants from 1147 events resistant to RTSV have one allele encoding eIF4G of nearly full length with in‐frame mutations in SVLFPNLAGKS residues located immediately upstream of the YVV residues, while the other allele encodes a truncated eIF4G with the YVV residues mutated to LCC (Figures 3b, 4). On the other hand, the reactions to RTSV of T_2_ plants derived from 1146 and 1148 events were inconsistent, showing resistance or susceptibility (Tables S4, S6). All of the T_2_ plants from 1146 and 1148 events have one allele encoding eIF4G of nearly full length with mutations located about 76 amino acids upstream of the YVV residues or immediately downstream of the YVV residues and possessing intact YVV residues (Figures 3b, 4).

**Figure 5:**
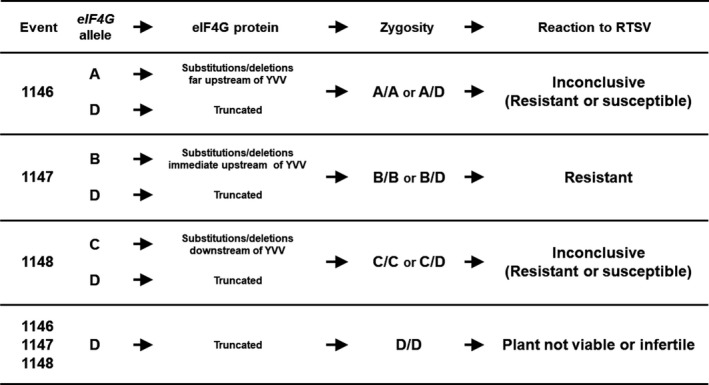
Reactions of the edited plants to RTSV depending on the type and zygosity of mutations. The novel *eIF4G* alleles are classified into four groups: A‐type allele resulting in substitutions/deletions about 76 amino acids upstream of the YVV residues; B‐type allele resulting in substitutions/deletions immediately upstream of the YVV residues; C‐type allele resulting in substitutions/deletions downstream of the YVV residues; and D‐type allele resulting in premature stop codon. The observed phenotypes are A/A and A/D—inconclusive (resistant or susceptible), B/B and B/D—resistant, C/C and C/D—inconclusive (resistant or susceptible), and D/D—plant not viable.

To better understand the relationship between mutated *eIF4G* alleles and the observed RTSV phenotype, we grouped the alleles with mutations in the following four categories: (i) A‐type alleles encode eIF4G with amino acid substitutions/deletions about 76 amino acids upstream of the YVV residues, as in the case of 1146 events derived from the pCas9‐eIF4G‐gRNA1 construct; (ii) B‐type alleles encode eIF4G with changes in NL residues, upstream of the YVV residues, as in the case of 1147 events derived from the pCas9‐eIF4G‐gRNA2 construct; (iii) C‐type alleles encode eIF4G with substitutions/deletions immediately downstream of the YVV residues, as in the case of 1148 event derived from the pCas9‐eIF4G‐gRNA3 construct; and (iv) D‐type alleles encode a truncated eIF4G. All T_2_ plants from 1147 events with a B/B or a B/D allele combination were resistant to RTSV (Figure 5, Data S1). On the other hand, the reactions to RTSV of T_2_ plants from 1146 and 1148 events carrying an A/A, A/D, C/C or C/D allele combination were inconclusive, showing resistance or susceptibility (Figure 5, Table S6, Data S1). As mentioned above, plants with a D/D allele combination are not likely to be viable, and we were not able to observe any T_2_ plants with a D/D allele combination.

To further confirm the stability of the trait, selected T_2_ events were advanced to the T_3_ generation and selected T_3_ plants were evaluated for reactions to RTSV. All T_3_ plants from 1147 events were resistant, whereas the reactions of T_3_ plants derived from 1146 and 1148 events were still inconclusive, showing susceptibility or resistance to RTSV (Table S7). Sequencing analysis for the *eIF4G* alleles in the T_3_ plants from 1147 events showed that all of the plants have either B/B or B/D alleles (Table S8). Collectively, these results indicate that, among the mutated *eIF4G* alleles generated by the CIRSPR/Cas9 system, only the *eIF4G* alleles resulting in in‐frame mutations in the SVLFPNLAGKS sequence (mainly the NL residues) immediately upstream of the YVV residues can explicitly confer resistance to RTSV.

Agronomic parameters such as plant height, numbers of panicles and filled grains, and total filled‐grain weight were measured in the RTSV‐inoculated T_2_ plants from 1147 events and RTSV‐inoculated and mock‐inoculated IR64 plants (Table 2, Figure S5). When T_2_ plants from 1147 events were inoculated with RTSV, their agronomic parameters were significantly higher than those of the RTSV‐inoculated IR64 control plants under glasshouse conditions.

**Table 2:** Agronomic parameters measured in selected T_2_ plants grown under glasshouse conditions

T_1_ events	No. of analysed T_2_ plants	Plant height (cm)†	No. of panicles†	No. of filled grains†	Total filled grains weight (g)†
1147‐03‐10	20	70.3 ± 10.0	5.3 ± 2.3**	164.6 ± 103.9*	3.6 ± 2.3
1147‐10‐12	13	79.4 ± 8.5	5.4 ± 2.6**	244.3 ± 196.1**	5.5 ± 4.5**
1147‐10‐19	12	89.4 ± 5.0***	5.3 ± 1.6**	251.3 ± 118.1***	5.7 ± 2.7***
1147‐10‐20	15	78.1 ± 7.2	5.6 ± 2.2**	182.7 ± 117.9*	4.3 ± 2.8*
1147‐11‐07	22	84.3 ± 7.5**	5.0 ± 2.3**	186.7 ± 118.7*	4.4 ± 2.8*
1147‐13‐10	17	80.7 ± 5.2	6.9 ± 2.7	254.1 ± 127.4***	6.0 ± 3.0***
IR64 WT RTSV‡	15	80.9 ± 7.3	7.5 ± 3.2	119.5 ± 96.0	2.8 ± 2.3
IR64 WT CTRL§	8	92.2 ± 3.2***	4.2 ± 0.8**	215.0 ± 68.0**	4.9 ± 1.6**

^†^Data are expressed as means ± standard deviation. Results were subjected to analysis of variance, and the means were compared by Student's *t*‐test (*P <* *0.05, **P <* *0.01, ***P <* *0.001) using IR64 WT RTSV as the reference control. ^‡^IR64 WT RTSV represents the nontransgenic control subjected to RTSV inoculation. ^§^IR64 WT CTRL represents the nontransgenic untreated control (mock‐treated).

## Discussion

The development of rice varieties with resistance to RTD has been a major objective for breeding programmes in tropical Asian countries. Genotyping and phenotyping for RTD resistance of a great number of rice accessions have been carried out in the last 50 years (Azzam *et al*., 2000). The information gathered from previous studies on RTD resistance and the technical advances provided by the development of genome editing tools had set the stage for the current study.

Plant viruses need host factors to propagate their genomes in host cells. This dependence can be harnessed to develop crops with virus resistance. To this end, an effective approach is to produce targeted mutations in genes that encode a host factor critical for the viral infection process. Genome editing is a useful tool that can shorten the time needed to replace alleles and develop plants with specific traits (Puchta, 2017; Weeks *et al*., 2016; Xiong *et al*., 2015). In our case, the development of RTSV‐resistant rice was achieved in a considerably shorter period than with traditional breeding. The *eIF4G* gene targeted in this study is responsible for resistance to RTSV (Lee *et al*., 2010). Using three different gRNAs’ targeting regions surrounding the nucleotide sequence encoding the YVV residues associated with RTSV resistance (Lee *et al*., 2010), we obtained novel *eIF4G* alleles with high efficiency. The types of mutations obtained in T_0_ events, zygosity and transmissibility of the mutations to successive generations are in agreement with other studies performed in rice (Li *et al*., 2017; Miao *et al*., 2013; Xu *et al*., 2015; Zhang *et al*., 2014). The distorted segregation pattern of mutations (1:2:0 or 1:1:0) in the T_2_ generation could be explained, at least in part, by the presence of alleles that result in truncation of eIF4G. The eIF4G function seems to be essential for plant development, and homozygotes for alleles encoding a truncated eIF4G are most likely to be lethal as we did not observe T_2_ or T_3_ plants with homozygous alleles for a truncated eIF4G. This finding provides a new insight into the function of rice *eIF4G*. Albeit the presence of additional genes for eIF4G isoforms in the rice genome, which are thought to have a partial functional redundancy enabling to compensate the loss of eIF4G (Sanfaçon, 2015), our study shows that this is not likely to be the case. Finally, the T_2_ mutant plants no longer contained Cas9 transgene and no off‐target effects were observed, which are important criteria to avoid the regulatory constraints in commercialization/release of transgenic plants (Huang *et al*., 2016; Voytas and Gao, 2014).

Lee *et al*. (2010) and Leung *et al*. (2015) mentioned that the YVV residues are critical to determining whether eIF4G is associated with susceptibility or nonsusceptibility (resistance) to RTSV. The most interesting observation revealed in our study is that, as seen in RTSV‐resistant T_2_ and T_3_ plants from 1147 events, mutations within the SVLFPNLAGKS sequence (mainly the NL residues) located immediately upstream of the YVV residues appear to be associated with the reaction to RTSV. All RTSV‐resistant T_2_ and T_3_ plants from 1147 events can be categorized in two classes: (i) plants with the homozygous *eIF4G* allele containing in‐frame mutations that result in changes in at least two of the amino acid residues in SVLFPNLAGKS (e.g. NL residues) and (ii) biallelic plants with one allele containing in‐frame mutations resulting in changes in NL residues and the second allele containing mutations resulting in premature termination of eIF4G (Data S1). This finding underlines the presence of additional amino acid residues associated with RTSV resistance that can be modified to create novel *eIF4G* alleles conferring resistance to RTSV and thus broadening the range of possibilities for developing RTSV‐resistant varieties. On the other hand, the reaction to RTSV of the T_2_ plants from 1146 and 1148 events was inconclusive (Tables S4, S6, Data S1). The in‐frame mutations found in the T_2_ plants from the 1146 events and those from the 1148 events occurred further upstream or immediately downstream of the YVV (and SVLFPNLAGKS) residues. The inconclusive reactions could be a consequence of (i) minor effects on the reactions to RTSV by the mutations, (ii) moderate GLH resistance of IR64 that might prevent successful transmission of RTSV (Shibata *et al*., 2007; Zenna *et al*., 2008) or (iii) combination of both. In any case, the amino acids mutated in a nearly full‐length eIF4G of the T_2_ plants from 1146 and 1148 events are most likely not to have a significant effect on the reactions to RTSV.

The amino acid residues most commonly mutated in eIF4G of the RTSV‐resistant T_2_ plants from 1147 events were the NL residues within the SVLFPNLAGKS sequence. Thus, in addition to the YVV residues, the NL residues are likely to be other key residues determining the reaction of rice to RTSV. The biological function of SVLFPNLAGKSYVV residues in determining the resistance of rice plants to RTSV is still to be investigated. It was reported that mutations in the MIF4G domain (Ponting, 2000) of eIF(iso)4G, without affecting the structure of MIF4G, resulted in the loss of interaction between eIF(iso)4G and the VPg (viral protein genome‐liked) protein of RYMV and, subsequently, the loss of susceptibility of rice to RYMV (Hébrard *et al*., 2010). The SVLFPNLAGKSYVV residues in the rice eIF4G are located between the eIF4E‐binding motif (Mader *et al*., 1995) and the MIF4G domain, both of which are essential to the function of eIF4G. Therefore, it is reasonable to conjecture that the SVLFPNLAGKSYVV residues are potentially involved in the interaction between eIF4G and an RTSV protein that affects the reaction of rice to RTSV.

In summary, here we report on the development of IR64‐derived rice lines with resistance to RTSV by CRISPR/Cas9‐mediated targeted mutagenesis of *eIF4G*. The induced mutations were successfully transmitted to the next generations. As a recessive trait, homozygous lines are required for resistance, but in our study, biallelic mutants where only one functional mutated allele was present and the other allele encodes a truncated nonfunctional protein also showed the desired phenotype. Moreover, our results demonstrated that, in addition to the YVV residues (Lee *et al*., 2010; Leung *et al*., 2015), the NL residues within the SVLFPNLAGKS sequence of eIF4G are also critical to determine the reaction to RTSV and that in‐frame mutations in this sequence resulted in resistance to RTSV. The final products show resistance to RTSV without detectable off‐target mutations or Cas9 insertion in the genome. Therefore, the RTSV‐resistant plants generated in this study may represent a valuable material as alternative sources of RTSV resistance and may be released in RTD‐prone areas as potentially nongenetically modified varieties.

## Experimental procedure

### Design of CRISPR/Cas9 vectors

The IR64 *eIF4G* sequence was obtained from the SNP‐seek database (Mansueto *et al*., 2017). The gRNA targets in the *eIF4G* gene were selected using the E‐CRISP Design Tool (Heigwer *et al*., 2014). The Cas9 expression vectors were constructed as described in Čermák *et al*. (2017) using protocols 2A and 5. A version of pMOD_A1110 (ZmUbi:TaCas9—codon‐optimized for wheat) with the octopine synthase terminator and an additional NLS and a FLAG tag at the C terminus of Cas9, along with pMOD_B2518 (TaU6:gRNA), pMOD_C0000 (empty), and the pTRANS_240 transformation backbone (T‐DNA, 2x35S:hptII w/intron for selection) were used. The gRNA oligonucleotides were synthesized by Integrated DNA Technologies (Coralville, Iowa) (Table S1). The resulting vectors (pCas9‐eIF4G‐gRNA1, pCas9‐eIF4G‐gRNA2 and pCas9‐eIF4G‐gRNA3) were sequence‐verified (Macrogen, Seoul, Korea) to confirm the presence of all elements.

### *Agrobacterium*‐mediated transformation

The pCas9‐eIF4G‐gRNA1, pCas9‐eIF4G‐gRNA2 and pCas9‐eIF4G‐gRNA3 constructs were introduced into the virulent LBA4404 strain of *Agrobacterium tumefaciens* using the freeze/thaw method (Höfgen and Willmitzer, 1988). Immature embryos of *Oryza sativa* var. *indica* cv. IR64 were used for the *Agrobacterium*‐mediated transformation, as previously described (Slamet‐Loedin *et al*., 2014). For each construct, 50 immature embryos were used per transformation. The resulting regenerated plantlets were acclimatized to glasshouse conditions. The T_0_ seeds were harvested and used to generate the T_1_ population. Subsequently, T_1_ seeds were used to generate the T_2_ population and T_2_ seeds to generate the T_3_ population.

### Detection of mutations

Genomic DNA was extracted from wild‐type (WT) IR64, T_0_ events and plants of subsequent generations (T_1_, T_2_ and T_3_) using the CTAB method as previously described (Stewart and Via, 1993). To identify the presence of the T‐DNA in the rice genome, PCR was conducted using primers for *hyg* (Hyg F1 and Hyg R1), while the presence/absence of Cas9 was assessed using the primer pair Tacas9_1F and Tacas9_1R (Table S9). The PCR conditions were as follows: denaturation at 95 °C/5 min; 35 cycles of 95 °C/30 s, 55 °C/30 s and 72 °C/2 min; and a final extension at 72 °C/5 min. Positive transgenic events were screened for mutations using the T7EI assay. As a first step, PCR products (of about 1.5 kb) encompassing the gRNA target sites were generated using the genomic DNA as template with primers eIF4GtF and eIF4GtR (Table S9) and Q5 DNA polymerase (New England Biolabs (Ipswich, Massachusetts)). The PCR conditions were as follows: denaturation at 95 °C/5 min; 35 cycles of 95 °C/30 s, 55 °C/30 s and 72 °C/2 min; and a final extension step at 72 °C/5 min. The PCR products were purified using the QIAquick PCR Purification Kit (Qiagen, Hilden, Germany). In the second step, 200 ng of purified PCR products were denatured and re‐annealed in a PCR machine, and consecutively digested with 1U of T7 endonuclease I (New England Biolabs); the conditions used were as follows: 95 °C/5 min, 85 °C/5 min, 25 °C/5 min and 37 °C/1 h. As a last step, 0.25 μm EDTA was added to stop the reaction and the fragments were subjected to electrophoresis on an 1% agarose gel. Three independent PCRs for each positive sample were sequenced directly without cloning (Macrogen). The sequences were analysed using DSDecodeM (http://skl.scau.edu.cn/dsdecode/) to decode the superimposed sequencing chromatograms and CRISPR‐ID (http://crispid.gbiomed.kuleuven.be/) to detect the size and localization of indels.

### Off‐target analysis

CRISPR‐P (http://cbi.hzau.edu.cn/crispr/) was used to predict potential off‐target sites for each gRNA (Lei *et al*., 2014). The highest‐scoring off‐target (OFF1, chr 11: 17936212) was amplified from the genomic DNA of selected T_2_ plants from the 1147 events with primers CC_Target_F1 and CC_Target_R1 (Table S9). The resulting PCR products were analysed for mutations by Sanger sequencing (Macrogen).

### RTSV inoculation and detection

Candidate T_2_ and T_3_ progenies were inoculated with RTSV using GLH. In brief, three adult GLH males carrying RTSV strain A were allowed to feed on a 10‐day‐old plant for 24 h using the tube method as previously described (Cabauatan *et al*., 1995). After insect removal, the inoculated plants were maintained in the glasshouse. Rice cultivar TW16 was used as RTSV‐resistant control, while varieties Taichung Native 1 and IR64 were used as susceptible controls (Lee *et al*., 2010). Inoculated plants were evaluated for virus infection by ELISA at 21 days postinoculation (dpi) as described by Cabauatan *et al*. (1995) using the extracts from the second youngest leaf collected from each plant. The presence of RTSV in the leaf extract was determined by measurement of the absorbance at 405 nm using an ELISA reader (BioTek Instruments, Winooski, Vermont)). Samples with an absorbance value >0.1 were considered positive for RTSV infection.

### Agronomic parameters

T_2_ plants from selected T_1_ seeds were inoculated with RTSV and grown in the glasshouse until maturity to determine several agronomic parameters. Agronomic phenotypes such as plant height, panicle count, filled‐grain number and total filled‐grain weight were measured upon harvest. Plant height was measured from the base of the stalk to the tip of the highest panicle of the highest tiller. Seed count and seed weight were acquired upon processing in the transgenic seed laboratory. Data are expressed as means ± standard deviation values. Results were subjected to statistical analysis by Student's *t*‐test (*P <* *0.05, **P <* *0.01, ***P <* *0.001).

## Authors’ contributions

PCM and IRC conceptualized the study and designed the experiments; TC and DFV designed the vectors; CC generated and analysed the T_0_ population; ISL supervised the plant transformation and tissue culture; IRC and GBJ supervised and performed the RTSV phenotyping; NRS generated and characterized the T_1_, T_2_ and T_3_ populations; and AM, PCM and IRC analysed the data and wrote the manuscript. All authors reviewed the manuscript.

## Conflict of interest

The authors declare no competing financial interest.

## Supporting information


**Figure S1** Screening for mutations in T_0_ population. (a) Gel images of PCR reactions using primers (eIF4GtF and eIF4GtR) flanking the targeted region. (b) Gel images of T7E assay performed on amplified bands. Numbers in red represent samples positive for mutations as evidenced by the presence of double bands in T7E assay. L, 1 kb ladder; Ctrl, controls; C+, positive control (wild‐type DNA samples); C−, negative control (no DNA control); C + d, wild‐type digested; C + n, wild‐type nondigested
**Figure S2** Representative chromatograms of selective events from T_0_ population. The sequencing results provided by Macrogen were analyzed using DSDecodeM (http://skl.scau.edu.cn/dsdecode/) to decode the superimposed sequencing chromatograms, and CRISPR‐ID (http://crispid.gbiomed.kuleuven.be/) to detect the size and localization of indels
**Figure S3** Example of a T_0_ event (1148‐16) with a homozygous allele encoding a truncated eIF4G. Even if the YVV sequence is not affected, the truncated protein from both alleles resulted in a sterile plant
**Figure S4** Examination for the presence of mutations in the putative off‐target sites. The analysis was conducted in T_2_ generation derived from 1147 (gRNA2) lines. Three different putative off‐target sites were picked up by CRISPR‐P (http://cbi.hzau.edu.cn/crispr/) in an *in silico* analysis. The most probable off‐target site (OFF1) was selected to confirm the *in silico* results by PCR and Sanger sequencing. The alignment between WT and selected T_2_ mutated events (homozygous and biallelic) is shown and the putative target is evidenced in red
**Figure S5** Selected 90‐day‐old T_2_ plants at 80‐days post‐inoculation with RTSV. Ten‐day‐old mutated plants and respective controls (IR64 WT, non‐transformed TW16) were inoculated with RTSV via GLH and subsequently grown under greenhouse conditions
**Table S1** Analysis of gRNA sequences used for CRISPR/Cas9 vector construction
**Table S2** Type of mutations in T_0_ events obtained by transformation with three CRISPR/Cas9 constructs
**Table S3** Percentage of deletions, insertions, and substitutions found in T_0_ events
**Table S4** Type of mutations transmitted to T_1_ and T_2_ plants, and reactions to RTSV of T_2_ plants derived from T_0_ events generated by the three CRISPR/Cas9 constructs
**Table S5** Pattern of mutation transmissibility in T_1_ and T_2_ populations
**Table S6** RTSV phenotype of selected lines from T_2_ population. TW16 and Taichung Native 1 (TN1) were used as the resistant and susceptible controls, respectively
**Table S7** RTSV phenotype of selected lines from T_3_ population. TW16 and Taichung Native 1 (TN1) were used as the resistant and susceptible controls, respectively
**Table S8** Genotype of *eIF4G* and reaction to RTSV in selected T_3_ plants
**Table S9** Primer sequences used in the present study

**Data S1** Genotype and reaction to RTSV of T2 lines. Nucleotide and protein alignments are presented compared to IR64 wild type eIF4G sequence. R, resistant; S, susceptible; A, A‐type allele resulting in substitutions/deletions about 76 amino acids upstream of the YVV residues; B, B‐type allele resulting in substitutions/deletions immediately upstream of the YVV residues; C, C‐type allele resulting in substitutions/deletions downstream of the YVV residues; D, D‐type allele resulting in premature stop codon. The observed phenotypes are: A/A and A/D ‐ inconclusive (resistant or susceptible), B/B and B/D ‐ resistant, C/C and C/D ‐ inconclusive (resistant or susceptible)

## References

[pbi12927-bib-0001] Albar, L. , Bangratz‐Reyser, M. , Hébrard, E. , Ndjiondjop, M.N. , Jones, M. and Ghesquière, A. (2006) Mutations in the eIF(iso)4G translation initiation factor confer high resistance to rice yellow mottle virus. Plant J. 47, 417–426. 16774645 10.1111/j.1365-313X.2006.02792.x

[pbi12927-bib-0002] Anjaneyulu, A. , Satapathy, M.K. and Shukla, V.D. (1994) Rice Tungro. New Delhi, India: Oxford and IBH Publishing Co., Pvt. Ltd.

[pbi12927-bib-0003] Azzam, O. and Chancellor, T.C. (2002) The biology, epidemiology, and management of rice tungro disease in Asia. Plant Dis. 86, 88–100. 10.1094/PDIS.2002.86.2.88 30823328

[pbi12927-bib-0004] Azzam, O. , Cabunagan, R.C. and Chancellor, T. (2000) Methods for Evaluating Resistance to Rice Tungro Disease. IRRI Discussion Paper Series No. 38., p. 40 Makati City, Philippines: International Rice Research Institute.

[pbi12927-bib-0005] Azzam, O. , Imbe, T. , Ikeda, R. , Nath, P.D. and Coloquio, E. (2001) Inheritance of resistance to rice tungro spherical virus in a near‐isogenic line derived from Utri Merah and in rice cultivar TKM6. Euphytica, 122, 91–97.

[pbi12927-bib-0006] Barakate, A. and Stephens, J. (2016) An overview of CRISPR‐based tools and their implementations: new opportunities in understanding plant‐pathogen interactions for better crop production. Front. Plant Sci. 7, 765. 27313592 10.3389/fpls.2016.00765 PMC4887484

[pbi12927-bib-0007] Belhaj, K. , Chaparro‐Garcia, A. , Kamoun, S. and Nebraskov, K. (2013) Plant genome editing made easy: targeted mutagenesis in model and crop plants using the CRISPR/Cas system. Plant Methods, 11, 39. 10.1186/1746-4811-9-39 PMC3852272 24112467

[pbi12927-bib-0008] Cabauatan, P.Q. , Cabunagan, R.C. and Koganezawa, H. (1995) Biological variants of rice tungro viruses in the Philippines. Phytopathology, 85, 77–81.

[pbi12927-bib-0009] Cabunagan, R.C. , Angeles, E.R. , Villareal, S. , Jefferson, O. , Teng, P.P.S. , Khush, G.S. , Chancellor, T.C.B. *et al* (1999) Multilocation evaluation of advanced breeding lines for resistance to rice tungro viruses In Rice Tungro Disease Management, (Chancellor T.C.B., Azzam O. and Heong K.L., eds), pp. 45–55. Manila, Philippines: International Rice Research Institute.

[pbi12927-bib-0010] Čermák, T. , Curtin, S.J. , Gil‐Humanes, J. , Čegan, R. , Kono, T.J.Y. , Konečná, E. , Belanto, J.J. *et al* (2017) A multipurpose toolkit to enable advanced genome engineering in plants. Plant Cell, 29, 1196–1217. 28522548 10.1105/tpc.16.00922 PMC5502448

[pbi12927-bib-0011] Chancellor, T.C.B. , Holt, J. , Villareal, S. , Tiongco, E.R. and Venn, J. (2006) Spread of plant virus disease to new plantings: a case study of rice tungro disease. Adv. Virus Res. 66, 1–29. 16877058 10.1016/S0065-3527(06)66001-6

[pbi12927-bib-0012] Chandrasekaran, J. , Brumin, M. , Wolf, D. , Leibman, D. , Klap, C. , Pearlsman, M. , Sherman, A. *et al* (2016) Development of broad virus resistance in non‐transgenic cucumber using CRISPR/Cas9 technology. Mol. Plant Pathol. 17, 1140–1153. 26808139 10.1111/mpp.12375 PMC6638350

[pbi12927-bib-0013] Dreher, T.W. and Miller, W.A. (2006) Translational control in positive strand RNA viruses. Virology, 344, 185–197. 16364749 10.1016/j.virol.2005.09.031 PMC1847782

[pbi12927-bib-0014] Ebron, L.A. , Yumol, R.R. , Ikeda, R. and Imbe, T. (1994) Inheritance of resistance to rice tungro spherical virus in some rice cultivars. Int. Rice Res. Notes, 19, 10–11.

[pbi12927-bib-0015] Encabo, J.R. , Cabauatan, P.Q. , Cabunagan, R.C. , Satoh, K. , Lee, J.H. , Kwak, D.Y. , De Leon, T.B. *et al* (2009) Suppression of two tungro viruses in rice by separable traits originating from cultivar Utri Merah. Mol. Plant‐Microbe. 22, 1268–1281. 10.1094/MPMI-22-10-1268 19737100

[pbi12927-bib-0016] Feng, Z.I. , Mao, Y. , Xu, N. , Zhang, B. , Wei, P. , Yang, D.L. , Wang, Z. *et al* (2014) Multigeneration analysis reveals the inheritance, specificity, and patterns of CRISPR/Cas‐induced gene modifications in *Arabidopsis* . Proc. Natl Acad. Sci. USA, 111, 4632–4637. 24550464 10.1073/pnas.1400822111 PMC3970504

[pbi12927-bib-0017] Hébrard, E. , Poulicard, N. , Gérard, C. , Traoré, O. , Wu, H.C. , Albar, L. , Fargette, D. *et al* (2010) Direct interaction between the Rice yellow mottle virus (RYMV) VPg and the central domain of the rice eIF(iso)4G1 factor correlates with rice susceptibility and RYMV virulence. Mol. Plant‐Microbe Interact. 23, 1506–1513. 20653414 10.1094/MPMI-03-10-0073

[pbi12927-bib-0018] Heigwer, F. , Kerr, G. and Boutros, M. (2014) E‐CRISP: fast CRISPR target site identification. Nat. Methods, 11, 122–123. 24481216 10.1038/nmeth.2812

[pbi12927-bib-0019] Hibino, H. and Cabauatan, P.Q. (1987) Infectivity neutralization of rice tungro associated viruses acquired by vector leafhoppers. Phytopathology, 77, 473–476.

[pbi12927-bib-0020] Hibino, H. , Daquioag, R.D. , Cabauatan, P.Q. and Dahal, G. (1988) Resistance to rice tungro spherical virus in rice. Plant Dis. 72, 843–847.

[pbi12927-bib-0021] Hibino, H. , Daquiaog, R.D. , Mesina, E.M. and Aguiero, V.M. (1990) Resistances in rice to tungro‐associated viruses. Plant Dis. 74, 923–926.

[pbi12927-bib-0022] Höfgen, R. and Willmitzer, L. (1988) Storage of competent cells for *Agrobacterium* transformation. Nucleic Acids Res. 16, 9877. 3186459 10.1093/nar/16.20.9877 PMC338805

[pbi12927-bib-0023] Huang, S. , Weigel, D. , Beachy, R.N. and Li, J. (2016) A proposed regulatory framework for genome‐edited crops. Nat. Genet. 48, 109–111. 26813761 10.1038/ng.3484

[pbi12927-bib-0024] Hull, R. (1996) Molecular biology of rice tungro viruses. Annu. Rev. Phytopathol. 34, 275–297. 15012544 10.1146/annurev.phyto.34.1.275

[pbi12927-bib-0025] Khush, G.S. , Angeles, E. , Virk, P.S. and Brar, D.S. (2004) Breeding rice for resistance to tungro virus at IRRI. SABRAO J. Breed. Genet. 36, 101–106.

[pbi12927-bib-0026] Kneller, E.L. , Rakotondrafara, A.M. and Millerb, W.A. (2006) Cap‐independent translation of plant viral RNAs. Virus Res. 119, 63–75. 16360925 10.1016/j.virusres.2005.10.010 PMC1880899

[pbi12927-bib-0027] Lee, J.H. , Muhsin, M. , Atienza, G.A. , Kwak, D.Y. , Kim, S.M. , De Leon, T.B. , Angeles, E.R. *et al* (2010) Single nucleotide polymorphisms in a gene for translation initiation factor (eIF4G) of rice (*Oryza sativa*) associated with resistance to rice tungro spherical virus. Mol. Plant‐Microbe Interact. 23, 29–38. 19958136 10.1094/MPMI-23-1-0029

[pbi12927-bib-0028] Lee, J. , Chung, J.H. , Kim, H.M. , Kim, D.W. and Kim, H. (2016) Designed nucleases for targeted genome editing. Plant Biotechnol. J. 14, 448–462. 26369767 10.1111/pbi.12465 PMC11389202

[pbi12927-bib-0029] Lei, Y. , Lu, L. , Liu, H.Y. , Li, S. , Xing, F. and Chen, L.L. (2014) CRISPR‐P: a web tool for synthetic single‐guide RNA design of CRISPR‐system in plants. Mol. Plant, 7, 1494–1496. 24719468 10.1093/mp/ssu044

[pbi12927-bib-0030] Leung, H. , Raghavan, C. , Zhou, B. , Oliva, R. , Choi, I.R. , Lacorte, V. , Jubay, M.L. *et al* (2015) Allele mining and enhanced genetic recombination for rice breeding. Rice, 8, 34. 10.1186/s12284-015-0069-y PMC4659784 26606925

[pbi12927-bib-0031] Li, J. , Sun, Y. , Du, J. , Zhao, Y. and Xia, L. (2017) Generation of targeted point mutations in rice by a modified CRISPR/Cas9 system. Mol. Plant, 10, 526–529. 27940306 10.1016/j.molp.2016.12.001

[pbi12927-bib-0032] Liang, G. , Zhang, H. , Lou, D. and Yu, D. (2016) Selection of highly efficient sgRNAs for CRISPR/Cas9‐based plant genome editing. Sci. Rep. 19, 21451. 10.1038/srep21451 PMC4759811 26891616

[pbi12927-bib-0033] Liu, X. , Homma, A. , Sayadi, J. , Yang, S. , Ohashi, J. and Takumi, T. (2016) Sequence features associated with the cleavage efficiency of CRISPR/Cas9 system. Sci. Rep. 27, 19675. 10.1038/srep19675 PMC4728555 26813419

[pbi12927-bib-0034] Mader, S. , Lee, H. , Pause, A. and Sonenberg, N. (1995) The translation initiation factor eIF‐4E binds to a common motif shared by the translation factor eIF‐4 gamma and the translational repressors 4E‐binding proteins. Mol. Cell. Biol. 15, 4990–4997. 7651417 10.1128/mcb.15.9.4990 PMC230746

[pbi12927-bib-0035] Mansueto, L. , Fuentes, R.R. , Borja, F.N. , Detras, J. , Abriol‐Santos, J.M. , Chebotarov, D. , Sanciangco, M. *et al* (2017) Rice SNP‐seek database update: new SNPs, indels, and queries. Nucleic Acids Res. 4, D1075–D1081. 10.1093/nar/gkw1135 PMC5210592 27899667

[pbi12927-bib-0036] Miao, J. , Guo, D. , Zhang, J. , Huang, Q. , Qin, G. , Zhang, X. , Wan, J. *et al* (2013) Targeted mutagenesis in rice using CRISPR‐Cas system. Cell Res. 23, 1233–1236. 23999856 10.1038/cr.2013.123 PMC3790239

[pbi12927-bib-0037] Muralidharan, K. , Krishnaveni, D. , Rajarajeshwari, N.V.L. and Prasad, A.S.R. (2003) Tungro epidemic and yield losses in paddy fields in India. Curr. Sci. 85, 1143–1147.

[pbi12927-bib-0038] Pan, C. , Ye, L. , Qin, L. , Liu, X. , He, Y. , Wang, J. , Chen, L. *et al* (2016) CRISPR/Cas9‐mediated efficient and heritable targeted mutagenesis in tomato plants in the first and later generations. Sci. Rep. 6, 24765. 27097775 10.1038/srep24765 PMC4838866

[pbi12927-bib-0039] Ponting, C.P. (2000) Novel eIF4G domain homologues linking mRNA translation with nonsense‐mediated mRNA decay. Trends Biochem. Sci. 25, 423–426. 10973054 10.1016/s0968-0004(00)01628-5

[pbi12927-bib-0040] Puchta, H. (2017) Applying CRISPR/Cas for genome engineering in plants: the best is yet to come. Curr. Opin. Plant Biol. 36, 1–8. 27914284 10.1016/j.pbi.2016.11.011

[pbi12927-bib-0041] Pyott, D.E. , Sheehan, E. and Molnar, A. (2016) Engineering of CRISPR/Cas9‐ mediated potyvirus resistance in transgene‐free *Arabidopsis* plants. Mol. Plant Pathol. 17, 1276–1288. 27103354 10.1111/mpp.12417 PMC5026172

[pbi12927-bib-0042] Rao, G.M. and Anjaneyulu, A. (1980) Estimation of yield losses due to tungro virus infection in rice cultivars. Oryza, 17, 210–214.

[pbi12927-bib-0043] Ronald, P.C. (2014) Lab to farm: applying research on plant genetics and genomics to crop improvement. PLoS Biol. 12, e1001878. 24915201 10.1371/journal.pbio.1001878 PMC4051633

[pbi12927-bib-0044] Sanfaçon, H. (2015) Plant translation factors and virus resistance. Viruses, 7, 3392–3419. 26114476 10.3390/v7072778 PMC4517107

[pbi12927-bib-0045] Sebastian, L.S. , Ikeda, R. , Hunag, N. , Imbe, T. , Coffman, W.R. and McCouch, S.R. (1996) Molecular mapping of resistance to rice tungro spherical virus and green leafhopper. Phytopathology, 86, 25–30.

[pbi12927-bib-0046] Seck, P.A. , Diagne, A. , Mohanty, S. and Woperies, M.C.S. (2012) Crops that feed the world 7: rice. Food Sec. 4, 7–24.

[pbi12927-bib-0047] Shan, Q. , Wang, Y. , Li, J. , Zhang, Y. , Chen, K. , Liang, Z. , Zhang, K. *et al* (2013) Targeted genome modification of crop plants using a CRISPR‐Cas system. Nat. Biotechnol. 31, 686–688. 23929338 10.1038/nbt.2650

[pbi12927-bib-0048] Shibata, Y. , Cabunagan, R.C. , Cabauatan, P.Q. and Choi, I.R. (2007) Characterization of *Oryza rufipogon*‐derived resistance to tungro disease in rice. Plant Dis. 91, 1386–1391. 10.1094/PDIS-91-11-1386 30780748

[pbi12927-bib-0049] Slamet‐Loedin, I. , Chadha‐Mohanty, P. and Torrizo, L. (2014) *Agrobacterium* mediated transformation: rice transformation. Methods Mol. Biol. 1099, 261–271. 24243210 10.1007/978-1-62703-715-0_21

[pbi12927-bib-0050] Sonenberg, N. and Hinnebusch, A.G. (2009) Regulation of translation initiation in eukaryotes: mechanisms and biological targets. Cell, 136, 731–745. 19239892 10.1016/j.cell.2009.01.042 PMC3610329

[pbi12927-bib-0051] Stewart, C.N. Jr and Via, L.E. (1993) A rapid CTAB DNA isolation technique useful for RAPD fingerprinting and other PCR applications. Biotechniques, 14, 748–750. 8512694

[pbi12927-bib-0052] Voytas, D.F. and Gao, C. (2014) Precision genome engineering and agriculture: opportunities and regulatory challenges. PLoS Biol. 12, e1001877. 24915127 10.1371/journal.pbio.1001877 PMC4051594

[pbi12927-bib-0053] Weeks, D.P. , Spalding, M.H. and Yang, B. (2016) Use of designer nucleases for targeted gene and genome editing in plants. Plant Biotechnol. J. 14, 483–495. 26261084 10.1111/pbi.12448 PMC11388832

[pbi12927-bib-0054] Xiong, J.S. , Ding, J. and Li, Y. (2015) Genome‐editing technologies and their potential application in horticultural crop breeding. Hortic. Res. 2, 15019. 26504570 10.1038/hortres.2015.19 PMC4595993

[pbi12927-bib-0055] Xu, R.F. , Li, H. , Qin, R.Y. , Li, J. , Qiu, C.H. , Yang, Y.C. , Ma, H. *et al* (2015) Generation of inheritable and “transgene clean” targeted genome‐modified rice in later generations using the CRISPR/Cas9 system. Sci. Rep. 19, 11491. 10.1038/srep11491 PMC5155577 26089199

[pbi12927-bib-0056] Zaidi, S.S. , Tashkandi, M. , Mansoor, S. and Mahfouz, M.M. (2016) Engineering plant immunity: using CRISPR/Cas9 to generate virus resistance. Front. Plant Sci. 7, 1673. 27877187 10.3389/fpls.2016.01673 PMC5099147

[pbi12927-bib-0057] Zenna, N.S. , Cabauatan, P.Q. , Baraoidan, M. , Leung, H. and Choi, I.R. (2008) Characterization of a putative rice mutant for reaction to rice tungro disease. Crop Sci. 48, 480–486.

[pbi12927-bib-0058] Zhang, H. , Zhang, J. , Wei, P. , Zhang, B. , Gou, F. , Feng, Z. , Mao, Y. *et al* (2014) The CRISPR/Cas9 system produces specific and homozygous targeted gene editing in rice in one generation. Plant Biotechnol. J. 12, 797–807. 24854982 10.1111/pbi.12200

[pbi12927-bib-0059] Zhu, C. , Bortesi, L. , Baysal, C. , Twyman, R.M. , Fischer, R. , Capell, T. , Schillberg, S. *et al* (2017) Characteristics of genome editing mutations in cereal crops. Trends Plant Sci. 22, 38–52. 27645899 10.1016/j.tplants.2016.08.009

