# Supplementary material for: Novel alleles of rice eIF4G generated by CRISPR/Cas9‐targeted mutagenesis confer resistance to Rice tungro spherical virus
Source: Plant Biotechnol J. 2018 Apr 30;16(11):1918–27. doi: 10.1111/pbi.12927 (PMC6181218; doi:10.1111/pbi.12927)
Supplement: Supplementary file 1 — Figure S1 Screening for mutations in T0 population. (a) Gel images of PCR reactions using primers (eIF4GtF and eIF4GtR) flanking the targeted region. (b) Gel images of T7E assay performed on amplified bands. Numbers in red represent samples positive for mutations as evidenced by the presence of double bands in T7E assay. L, 1 kb ladder; Ctrl, controls; C+, positive control (wild‐type DNA samples); C−, negative control (no DNA control); C + d, wild‐type digested; C + n, wild‐type nondigested Figure S2 Representative chromatograms of selective events from T0 population. The sequencing results provided by Macrogen were analyzed using DSDecodeM (http://skl.scau.edu.cn/dsdecode/) to decode the superimposed sequencing chromatograms, and CRISPR‐ID (http://crispid.gbiomed.kuleuven.be/) to detect the size and localization of indels Figure S3 Example of a T0 event (1148‐16) with a homozygous allele encoding a truncated eIF4G. Even if the YVV sequence is not affected, the truncated protein from both alleles resulted in a sterile plant Figure S4 Examination for the presence of mutations in the putative off‐target sites. The analysis was conducted in T2 generation derived from 1147 (gRNA2) lines. Three different putative off‐target sites were picked up by CRISPR‐P (http://cbi.hzau.edu.cn/crispr/) in an in silico analysis. The most probable off‐target site (OFF1) was selected to confirm the in silico results by PCR and Sanger sequencing. The alignment between WT and selected T2 mutated events (homozygous and biallelic) is shown and the putative target is evidenced in red Figure S5 Selected 90‐day‐old T2 plants at 80‐days post‐inoculation with RTSV. Ten‐day‐old mutated plants and respective controls (IR64 WT, non‐transformed TW16) were inoculated with RTSV via GLH and subsequently grown under greenhouse conditions Table S1 Analysis of gRNA sequences used for CRISPR/Cas9 vector construction Table S2 Type of mutations in T0 events obtained by transformation with three CRISPR/Cas9 co [file PBI-16-1918-s002.docx]

**SUPPORTING INFORMATION**

**Novel alleles of rice *eIF4G* generated by CRISPR/Cas9-targeted mutagenesis confer resistance to *Rice tungro spherical virus***

Anca Macovei^1,a,#^, Neah Rosandra Sevilla^1,#^, Christian Cantos^1,b^, Gilda Jonson^1^, Inez Slamet-Loedin^1^, Tomáš Čermák^2^, Dan Voytas^2^, Il-Ryong Choi^1^, Prabhjit Chadha-Mohanty^1,*^

^1^ Genetics and Biotechnology Division, International Rice Research Institute (IRRI), DAPO Box 7777, Metro Manila 1277, Philippines

^2^ Department of Genetics, Cell Biology & Development and Center for Genome Engineering, University of Minnesota, Minneapolis, Minnesota 55455, USA

** Corresponding Author:* PCM, e-mail: [pchadhamohanty@gmail.com](mailto:pchadhamohanty@gmail.com)

^#^ AM and NRS contributed equally to the work

Present address:

^a^ Department of Biology and Biotechnology ‘L. Spallanzani’, University of Pavia, via Ferrata 1, 27100 Pavia, Italy

^b^ Huck Institute of the Life Sciences, Pennsylvania State University, University Park, 16802 Pennsylvania, USA

**Table S1.** Analysis of gRNA sequences used for CRISPR/Cas9 vector construction.

|  | **Nucleotide sequence (5’-3’)^1^** | **Position^2^** | **PAM** | **GC^3^ (%)** | **N-20** | **N-3** | **Mutation rate^4^ (%)** |
| --- | --- | --- | --- | --- | --- | --- | --- |
| **gRNA1** | GGTTAATATGGGTGAGGACG | 4140-4158 | AGG | 50 | G | T | 36.0 |
| **gRNA2** | GTTCTGTTCCCTAATTTGGC | 4356-4375 | CGG | 45 | C | T | 86.6 |
| **gRNA3** | GATCACCCAAGTCCTGGAAG | 4395-4414 | GGG | 55 | G | T | 65.6 |

^1^DNA sequence representing gRNA.

^2^Nucleotide position in LOC_Os07g36940.

^3^IDT OligoAnalyzer 3.1 (<https://eu.idtdna.com/calc/analyzer>) was used to determine the GC%.

^4^Mutation rate is calculated as the number of total transgenic events containing mutations at the designated site/the number of total transgenic events.

**Table S2.** Type of mutations in T_0_ events obtained by transformation with three CRISPR/Cas9 constructs.

| **Event ID^1^** | **Type of mutations^2^** | **Zygosity** | **T-DNA** |
| --- | --- | --- | --- |
| 1146-06 | 21d/1s; 1i4s/15d2s; 1i6s/15d1s | Chimera | + |
| 1146-07 | 3d/1d | Biallelic | + |
| 1146-08 | 3d/3d2s; 3d1s/3d1s | Chimera | + |
| 1146-12 | 12d/4d; 12d/4d1s | Chimera | + |
| 1146-14 | 21d/1d1s; 21d/1d | Chimera | + |
| 1146-15 | 1d/4s1d; 1d2s/3s1d | Chimera | + |
| 1146-16 | 21d/1i | Biallelic | + |
| 1146-18 | 3d/1i | Biallelic | + |
| 1146-22 | 21d/5d1s; 21d/5d | Chimera | + |
| 1147-01 | 2d/WT | Biallelic | + |
| 1147-02 | 3d/WT | Biallelic | + |
| 1147-03 | 3d1s/1i; 3d1s/1i2s | Chimera | + |
| 1147-06 | 1i/3i; 3i/1i2s | Chimera | + |
| 1147-07 | 3d/1i | Biallelic | + |
| 1147-08 | 24d/1i | Biallelic | + |
| 1147-09 | 2s/1i; 1i/1i1d | Chimera | + |
| 1147-10 | 1i/12d | Biallelic | + |
| 1147-11 | 3d/1i | Biallelic | + |
| 1147-12 | 3d/1i | Biallelic | + |
| 1147-13 | 7d/WT | Biallelic | + |
| 1147-14 | 17d/WT | Biallelic | + |
| 1147-15 | 4d/6d | Biallelic | + |
| 1148-05 | 6d/4d | Biallelic | + |
| 1148-06 | 4d/4d1s; 4d/4d | Chimera | + |
| 1148-07 | 6d/2d2s | Biallelic | + |
| 1148-08 | 12d/1d | Biallelic | + |
| 1148-11 | 30d/2i | Biallelic | + |
| 1148-14 | 9d/4d | Biallelic | + |
| 1148-15 | 4d/2d1s | Biallelic | + |
| 1148-16 | 5d/5d | Homozygous | + |
| 1148-18 | 3d/1d | Biallelic | + |
| 1148-20 | 6d/4d | Biallelic | + |
| 1148-21 | 1d/3d1s; 3d/1d1s | Chimera | + |
| 1148-22 | 3d1s/2d1s; 3d/2d1s | Chimera | + |
| 1148-23 | 3d/8d; 8d/3d1s | Chimera | + |
| 1148-24 | 9d/4d; 4d/4d1s | Chimera | + |
| 1148-25 | 2d/2s | Biallelic | + |
| 1148-26 | 3d1s/3d1s | Biallelic | + |
| 1148-27 | 5d/3d1s; 3d2s/5d | Chimera | + |
| 1148-28 | 3d/2d | Biallelic | + |
| 1148-29 | 9d/4d | Biallelic | + |
| 1148-30 | 5d/3d | Biallelic | + |
| 1148-31 | 63d/4d | Biallelic | + |

^1^The 1146, 1147, and 1148 events were generated by the constructs containing DNA sequences representing gRNA1, gRNA2, and gRNA3, respectively.

^2^ d: deletion, i: insertion, s: substitution, and WT: wild type. The numbers in front of the letters indicate the number of nucleotides affected. Corresponding mutations in two alleles are distinguished by ‘/’. Different results in three PCR sequencing analysis, representative of chimeras, are distinguished by ‘;’.

**Table S3.** Percentage of deletions, insertions, and substitutions found in T_0_ events.

| **Vector ID** | **No. of events with mutations** | **Deletions (%)** | **Insertions (%)** | **Substitutions (%)** |
| --- | --- | --- | --- | --- |
| **1146** | 9 | 100.0 | 44.4 | 22.2 |
| **1147** | 12 | 83.3 | 25.0 | 58.3 |
| **1148** | 21 | 100.0 | 45.0 | 5.0 |

**Table S4.** Type of mutations transmitted to T_1_ and T_2_ plants, and reactions to RTSV of T_2_ plants derived from T_0_ events generated by the three CRISPR/Cas9 constructs.

| **Event ID** | **T_0_** | **T_1_** | | | **T_2_** | | | | |
| --- | --- | --- | --- | --- | --- | --- | --- | --- | --- |
|  | **Zygosity (mutation)^1^** | **Zygosity (mutation)^2^** | **Cas9^3^** | | **Zygosity (mutation)^2^** | **Cas9^3^** | | **RTSV^4^** | |
|  |  |  | **+** | **-** |  | **+** | **-** | **R** | **S** |
| 1146-12 | Chimera (12d/4d; 12d/4d1s) | 3 x biallelic (12d/4d) | 0 | 3 | 16 x homozygous (12d/12d) 15 x biallelic (12d/4d) | 0 | 31 | 12 | 19 |
|  |  |  |  |  |  |  |  |  |  |
| 1146-22 | Chimera (21d/5d1s; 21d/5d) | 2x biallelic (21d/5d1s) 2 x biallelic (21d/5d) | 2 | 2 | 7 x homozygous (21d/21d) 8 x biallelic (21d/5d) | 0 | 15 | 7 | 8 |
|  |  |  |  |  |  |  |  |  |  |
|  |  |  |  |  |  |  |  |  |  |
| 1147-03 | Chimera (3d1s/1i2s; 3d1s/1i) | 2 x biallelic (3d1s/1i) | 0 | 2 | 5 x biallelic (3d1s) 14 x biallelic (3d1s/1i) | 0 | 19 | 19 | 0 |
|  |  |  |  |  |  |  |  |  |  |
| 1147-10 | Biallelic (1i/12d) | 3 x biallelic (1i/12d) | 0 | 3 | 17 x homozygous (12d/12d) 15 x biallelic (1i/12d) | 0 | 32 | 32 | 0 |
|  |  |  |  |  |  |  |  |  |  |
| 1147-11 | Biallelic (3d/1i) | 2 x biallelic (3d/1i) | 1 | 1 | 7 x homozygous (3d/3d) 6 x biallelic (3d/1i) | 0 | 13 | 13 | 0 |
|  |  |  |  |  |  |  |  |  |  |
| 1147-13 | Biallelic (7d/WT) | 1 x biallelic (24d/1i2s) 1 x biallelic (24d/1i) | 1 | 1 | 9 x homozygous (24d/24d) 3 x biallelic (24d/1i2s)  1 x biallelic (24d/1i) | 0 | 13 | 13 | 0 |
|  |  |  |  |  |  |  |  |  |  |
| 1148-05 | Biallelic (6d/4d) | 1 x homozygous (6d/6d) 3 x biallelic (6d/4d) | 1 | 3 | 15 x homozygous (6d/6d) 16 x biallelic (6d/4d) | 0 | 31 | 9 | 22 |
|  |  |  |  |  |  |  |  |  |  |
| 1148-20 | Biallelic (6d/4d) | 4 x biallelic (6d/4d) | 2 | 2 | 8 x homozygous (6d/6d) 8 x biallelic (6d/4d) | 0 | 16 | 9 | 7 |
|  |  |  |  |  |  |  |  |  |  |

^1^d: deletion, i: insertion, s: substitution, and WT: wild type. The numbers in front of the letters indicate the number of nucleotides affected. Corresponding mutations in two alleles are distinguished by ‘/’. Different results in three PCR sequencing analysis, representative of chimeras, are distinguished by ‘;’.

^2^The numbers in front of zygosity are those of plants with the zygosity and the mutations indicated.

^3^Number of plants with (+) or without (-) the Cas9 sequence.

^4^Number of plants resistant (R) or susceptible (S).

**Table S5.** Pattern of mutation transmissibility in T_1_ and T_2_ populations.

| **T_0_ events** | **T_1_** | | | **T_2_** | | |
| --- | --- | --- | --- | --- | --- | --- |
|  | **No. of T_1_ lines** | **No. of T_1_ lines with mutations** | **Inheritance pattern^1^** | **No. of T_2_ lines** | **No. of T_2_ lines with mutations** | **Inheritance pattern^2^** |
| 1146-12 | 10 | 3 | 7:3 | 31 | 31 | 16:15:0 |
| 1146-22 | 9 | 4 | 5:4 | 15 | 15 | 7:8:0 |
| 1147-03 | 12 | 2 | 10:2 | 19 | 19 | 5:14:0 |
| 1147-10 | 20 | 13 | 7:13 | 32 | 32 | 17:15:0 |
| 1147-11 | 16 | 2 | 14:2 | 13 | 13 | 7:6:0 |
| 1147-13 | 9 | 3 | 6:3 | 13 | 13 | 9:3:1 |
| 1148-05 | 17 | 10 | 7:10 | 31 | 31 | 15:16:0 |
| 1148-20 | 8 | 5 | 3:5 | 16 | 16 | 8:8:0 |

^1^Inheritance pattern of T1 events is represented as number of plants without mutations : number of plants with mutations.

^2^The T_2_ inheritance pattern is represented as homozygous for mutation 1 : biallele : homozygous for mutation 2.

**Table S6.** RTSV phenotype of selected lines from T_2_ population. TW16 and Taichung Native 1 (TN1) were used as the resistant and susceptible controls, respectively.

| **Event ID/variety** | **Treatment** | **No. of samples tested** | **No. of infected samples** | **RTSV infection rate (%)** | **Reaction to RTSV^1^** |
| --- | --- | --- | --- | --- | --- |
| **Controls** | | | | | |
| TW16 | Mock | 5 | 0 | 0 | - |
| TN1 | Mock | 4 | 0 | 0 | - |
| IR64 | Mock | 5 | 0 | 0 | - |
| TW16 | RTSV | 10 | 0 | 0 | R |
| TN1 | RTSV | 10 | 7 | 70 | S |
| IR64 | RTSV | 9 | 7 | 78 | S |
| **IR64 CRISPR-generated T_2_ lines** | | | | | |
| 1146-12-02 | RTSV | 14 | 8 | 57 | I |
| 1146-12-05 | RTSV | 17 | 11 | 65 | S |
| 1146-22-11 | RTSV | 15 | 8 | 53 | I |
| 1147-03-10 | RTSV | 19 | 0 | 0 | R |
| 1147-10-12 | RTSV | 12 | 0 | 0 | R |
| 1147-10-19 | RTSV | 10 | 0 | 0 | R |
| 1147-10-20 | RTSV | 10 | 0 | 0 | R |
| 1147-11-07 | RTSV | 13 | 0 | 0 | R |
| 1147-13-10 | RTSV | 13 | 0 | 0 | R |
| 1148-05-15 | RTSV | 16 | 8 | 50 | I |
| 1148-05-17 | RTSV | 15 | 13 | 87 | S |
| 1148-20-03 | RTSV | 16 | 7 | 44 | I |

^1^Resistant (R), susceptible (S), and inconclusive (I) are based on a scale of infection 0-100%, where R represents 0-30%, I represents 31-60%, and S represents 61-100%.

**Table S7.** RTSV phenotype of selected lines from T_3_ population. TW16 and Taichung Native 1 (TN1) were used as the resistant and susceptible controls, respectively.

| **Event ID/ Variety** | **Treatment** | **Number of samples tested** | **Number of infected samples** | **RTSV infection rate (%)** | **Reaction to RTSV^1^** |
| --- | --- | --- | --- | --- | --- |
|  |  |  |  |  |  |
| **Controls** | | | | | |
| TW16 | Mock | 5 | 0 | 0 | - |
| TN1 | Mock | 5 | 0 | 0 | - |
| IR64 | Mock | 5 | 0 | 0 | - |
| TW16 | RTSV | 9 | 0 | 0 | R |
| TN1 | RTSV | 10 | 10 | 100 | S |
| IR64 | RTSV | 10 | 7 | 70 | S |
| **IR64 CRISPR-generated T_3_ lines** | | | | | |
| 1146-12-05-01 | RTSV | 20 | 12 | 60 | I |
| 1146-12-05-08 | RTSV | 19 | 14 | 74 | S |
| 1146-12-05-15 | RTSV | 20 | 13 | 65 | S |
| 1146-12-05-16 | RTSV | 19 | 10 | 53 | I |
| 1146-22-11-02 | RTSV | 20 | 13 | 65 | S |
| 1146-22-11-03 | RTSV | 20 | 14 | 70 | S |
| 1146-22-11-15 | RTSV | 18 | 12 | 67 | S |
| 1146-22-11-17 | RTSV | 20 | 13 | 65 | S |
| 1147-10-19-01 | RTSV | 20 | 0 | 0 | R |
| 1147-10-19-12 | RTSV | 19 | 0 | 0 | R |
| 1147-10-19-15 | RTSV | 20 | 0 | 0 | R |
| 1147-10-19-19 | RTSV | 20 | 0 | 0 | R |
| 1148-20-03-03 | RTSV | 20 | 10 | 50 | I |
| 1148-20-03-06 | RTSV | 16 | 9 | 56 | I |
| 1148-20-03-15 | RTSV | 13 | 10 | 77 | S |
| 1148-20-03-17 | RTSV | 15 | 12 | 80 | S |
| 1148-26-03-05 | RTSV | 18 | 15 | 83 | S |
| 1148-26-03-14 | RTSV | 16 | 16 | 100 | S |

^1^Resistant (R), susceptible (S), and inconclusive (I) are based on a scale of infection 0-100%, where R represents 0-30%, I represents 31-60%, and S represents 61-100%.

**Table S8.** Genotype of *eIF4G* and reaction to RTSV in selected T_3_ plants.

| **T_3_ Event ID** | **T_2_** | | | **T_3_** | | | |
| --- | --- | --- | --- | --- | --- | --- | --- |
|  | **Mutation^1^** | **Zygosity** | **RTSV^2^** | **Mutation^1^** | **Zygosity** | **Genotype^3^** | **RTSV^2^** |
| 1147-10-19-01-07 | 12d | Homozygous | R | 12d | Homozygous | B/B | R |
| 1147-10-19-01-13 |  |  | R |  |  |  | R |
| 1147-10-19-19-04 | 12d | Homozygous | R | 12d | Homozygous | B/B | R |
| 1147-10-19-19-19 |  |  | R |  |  |  | R |
| 1147-10-19-12-01 | 1i/12d | Biallelic | R | 12d | Homozygous | B/B | R |
| 1147-10-19-12-08 |  |  | R | 1i/12d | Biallelic | B/D | R |
| 1147-10-19-12-11 |  |  | R | 12d | Homozygous | B/B | R |
| 1147-10-19-12-14 |  |  | R | 1i/12d | Biallelic | B/D | R |
| 1147-10-19-12-16 |  |  | R | 12d | Homozygous | B/B | R |
| 1147-10-19-15-02 | 1i/12d | Ballelici | R | 12d | Homozygous | B/B | R |
| 1147-10-19-15-05 |  |  | R | 12d | Homozygous | B/B | R |
| 1147-10-19-15-12 |  |  | R | 12d | Homozygous | B/B | R |
| 1147-10-19-15-17 |  |  | R | 12d | Homozygous | B/B | R |
| 1147-10-19-15-20 |  |  | R | 12d | Homozygous | B/B | R |

^1^d: deletion, i: insertion. The numbers in front of the letters indicate the number of base pairs affected. Corresponding mutations in two alleles are distinguished by ‘/’.

^2^Resistant (R) to RTSV.

^3^B, B-type allele resulting in substitutions/deletions immediately upstream of the YVV residues; D, D-type allele resulting in premature stop codon.

**Table S9.** Primer sequences used in the present study.

| **Primer name** | **Primer sequence** |
| --- | --- |
| gRNA1_top | 5’-GATTGGTTAATATGGGTGAGGACGG-3’ |
| gRNA1_bottom | 5’-GATTGGTTAATATGGGTGAGGACGG-3’ |
| gRNA2_top | 5’-GATTGGTTAATATGGGTGAGGACGG-3’ |
| gRNA2_bottom | 5’-AAAACGCCAAATTAGGGAACAGAAC-3’ |
| gRNA3_top | 5’-GATTGATCACCCAAGTCCTGGAAGG-3’ |
| gRNA3_bottom | GATTGATCACCCAAGTCCTGGAAGG-3’ |
| ZY105F | 5’-ACGTAAGGGATGACGCACA-3’ |
| Hyg F1 | 5’-TCCGACCTGATGCAGCTCTC-3’ |
| Hyg R1 | 5’-GATTCCTTGCGGTCCGAATG-3’ |
| eIF4GtF | 5’-GCTCGACCAGTTTCAAGGGA-3’ |
| eIF4GtR | 5’-ACCCCAGTAAGAGTTGCCAC-3’ |
| CC Target F1 | 5’-AGTCTTCCTATCTTGTACTGAT-3’ |
| CC Target_R1 | 5’-GAACAACTCATACTTACACATC-3’ |
| Tacas9_1_F | 5’-GCTGATCACGCAAAGGAAGT-3’ |
| Tacas9_1_R | 5’-TTCTTTCTGGCGATGAGCTT-3’ |

**Figure S1.** Screening for mutations in T_0_ population. (**A**) Gel images of PCR reactions using primers (eIF4GtF and eIF4GtR) flanking the targeted region. (**B**) Gel images of T7E assay performed on amplified bands. Numbers in red represent samples positive for mutations as evidenced by the presence of double bands in T7E assay. L, 1 kb ladder; Ctrl, controls; C+, positive control (wild-type DNA samples); C-, negative control (no DNA control); C+d, wild-type digested; C+n, wild-type nondigested.

**
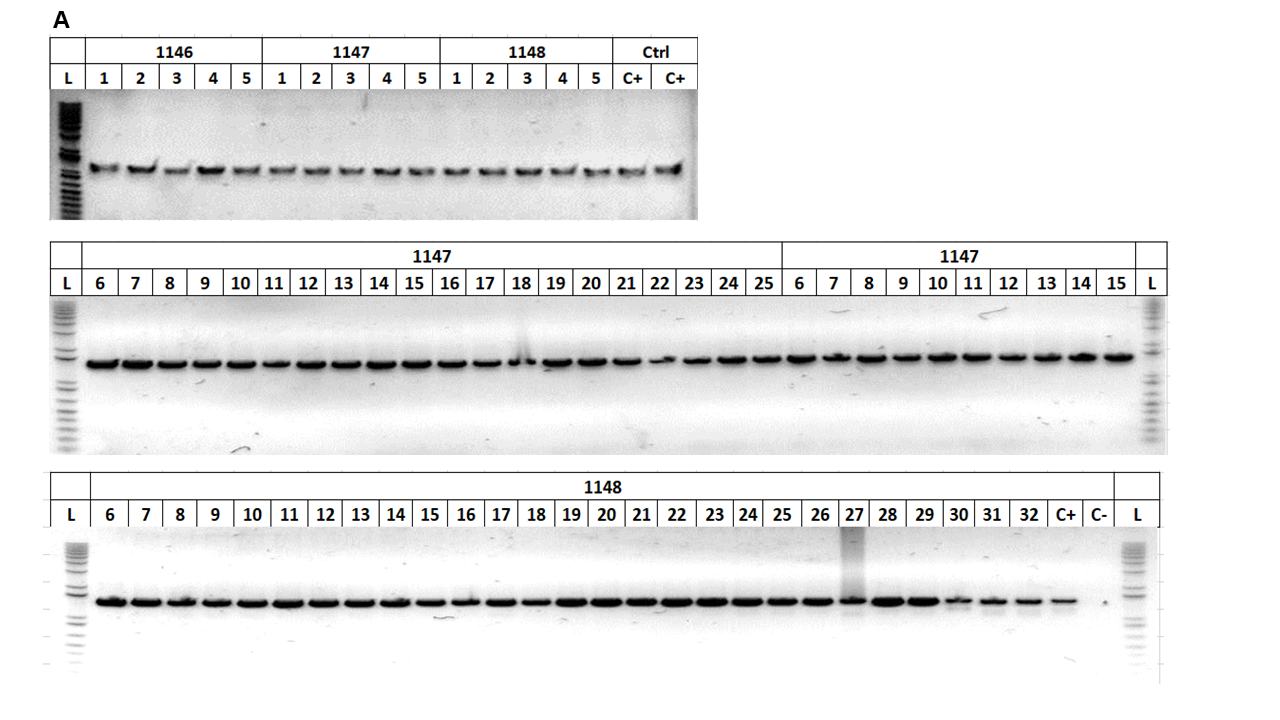
**

**
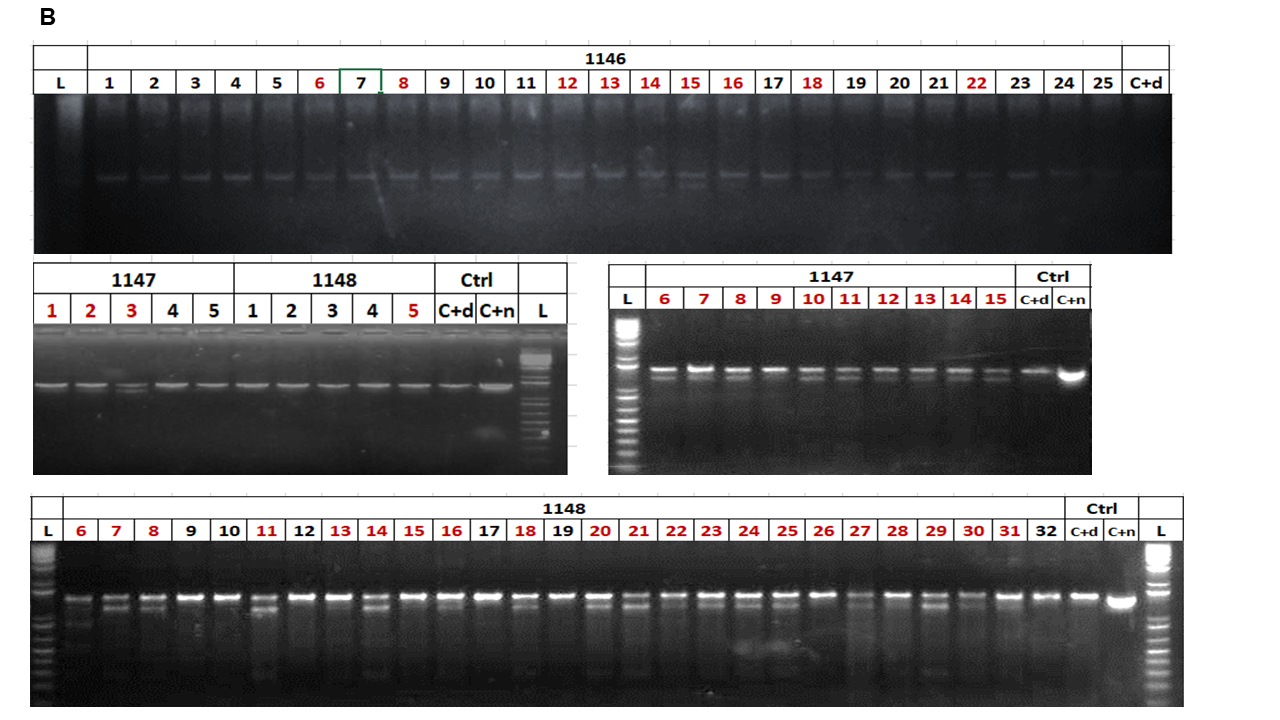
**

**Figure S2.** Representative chromatograms of selective events from T_0_ population. The sequencing results provided by Macrogen (Korea) were analyzed using DSDecode (http://dsdecode.scgene.com/) to decode the superimposed sequencing chromatograms, and CRISPR-ID (http://crispid.gbiomed.kuleuven.be/) to detect the size and localization of indels.


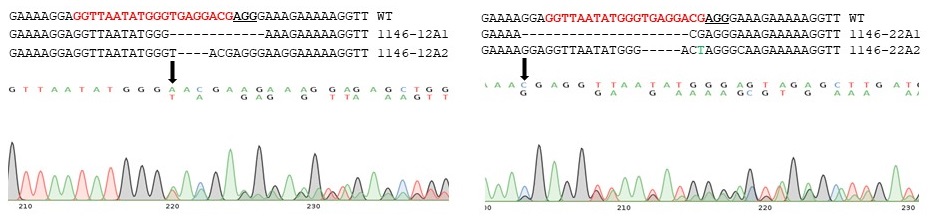


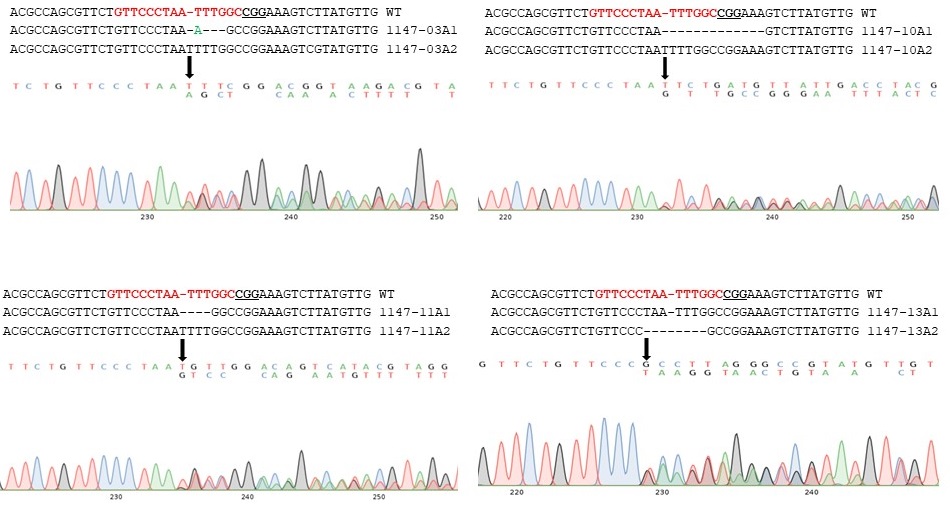


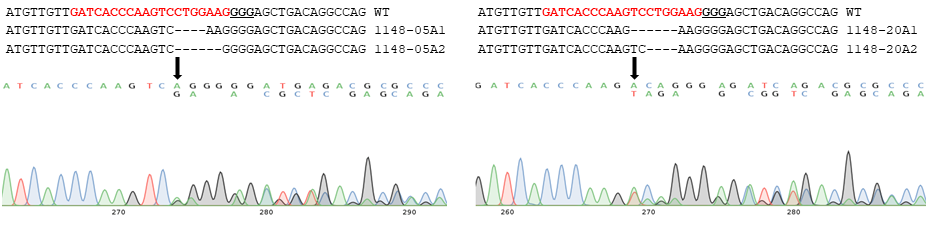


**Figure S3.** Example of a T_0_ event (1148-16) with a homozygous allele encoding a truncated eIF4G. Even if the YVV sequence is not affected, the truncated protein in both alleles resulted in a sterile plant.


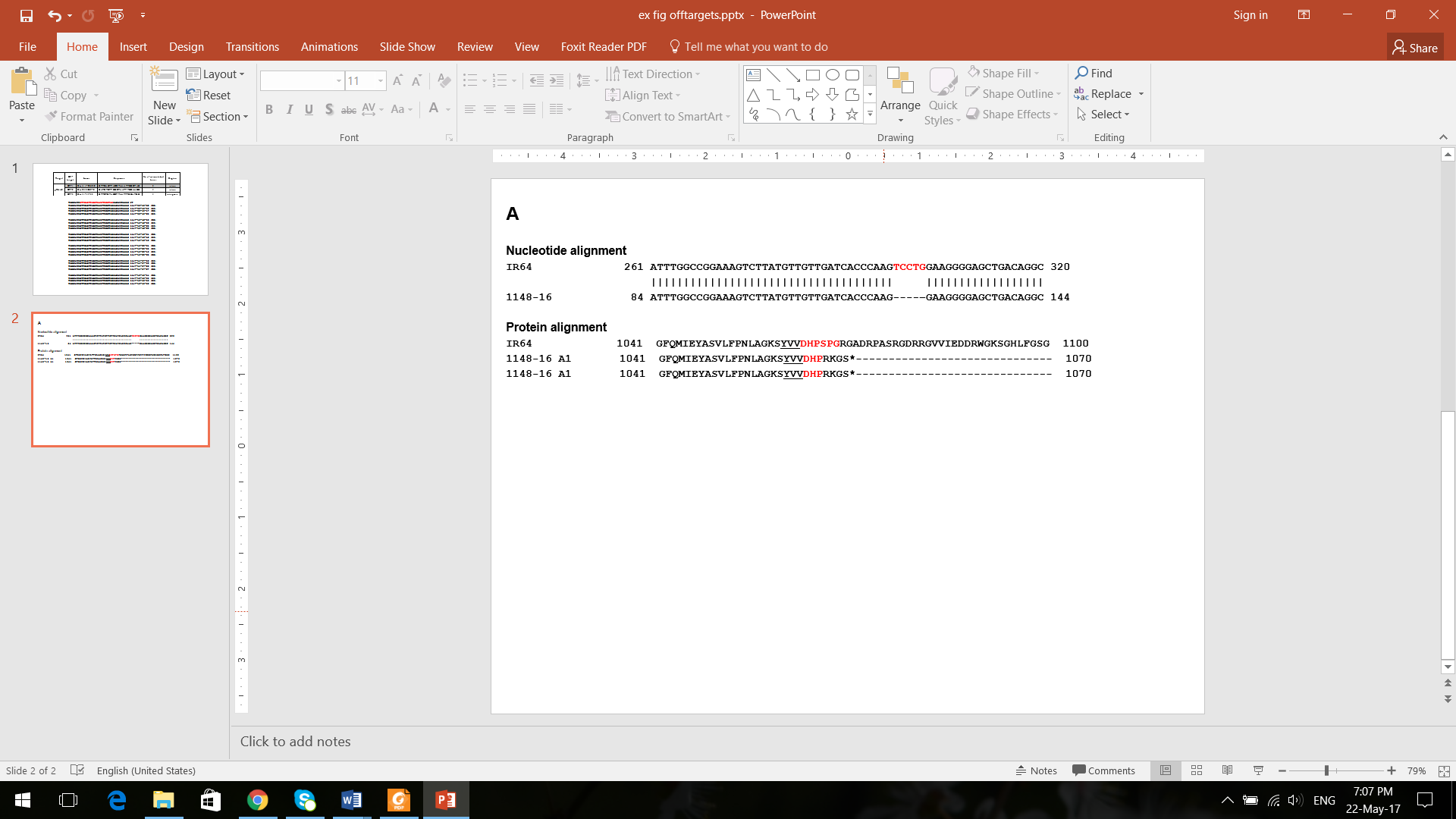


**Figure S4.** Examination for the presence of mutations in the putative off-target sites. The analysis was conducted in T_2_ generation derived from 1147 (gRNA2) lines. Three different putative off-target sites were picked up by CRISPR-P (<http://cbi.hzau.edu.cn/crispr/>) in an *in silico* analysis. The most probable off-target site (OFF1) was selected to confirm the *in silico* results by PCR and Sanger sequencing. The alignment between WT and selected T_2_ mutated events (homozygous and biallelic) is shown and the putative target is evidenced in red.

**
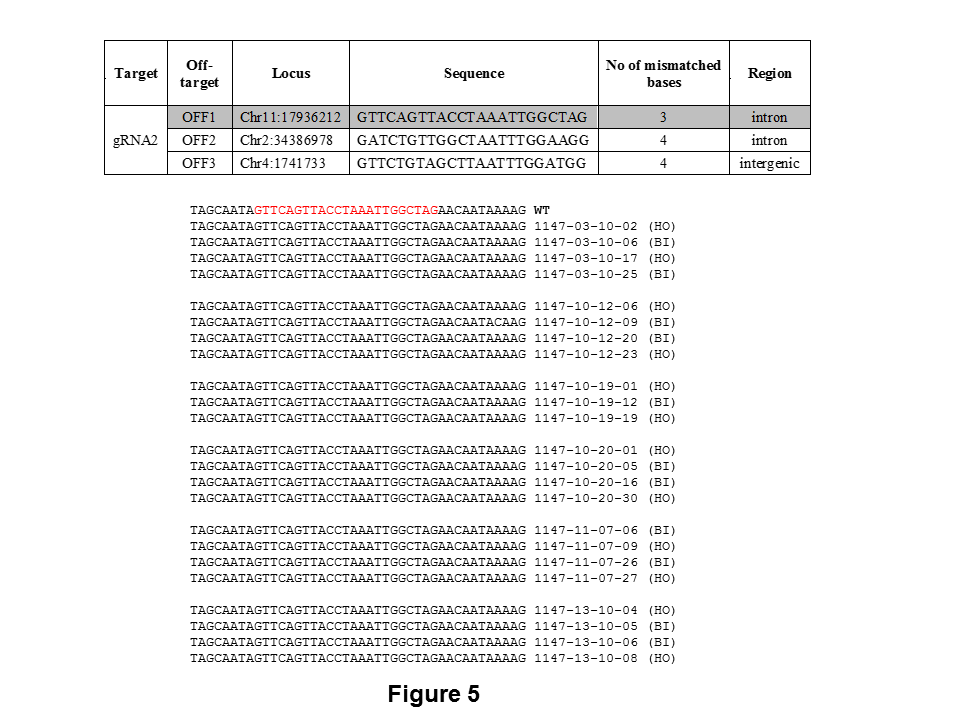
**

**Figure S5.** Selected 90-day-old T_2_ plants at 80-days post-inoculation with RTSV. Ten-day-old mutated plants and respective controls (IR64 WT, non-transformed TW16) were inoculated with RTSV via GLH and subsequently grown under greenhouse conditions.

**
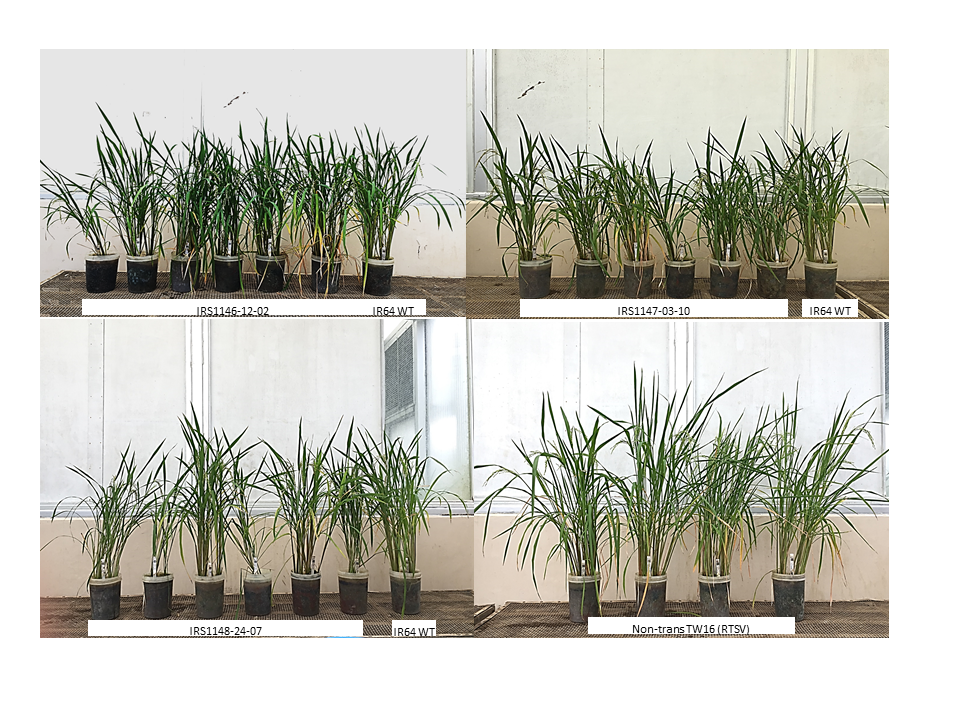
**
